# Bioarchaeological analysis illustrates the life of a 16th-century Sámi individual from Kitka, Kuusamo, northern Finland

**DOI:** 10.1186/s12864-026-12962-x

**Published:** 2026-05-25

**Authors:** Sanni Peltola, Ulla Nordfors, Laura Arppe, Markku Oinonen, Mika Sarkkinen, Miikka Voutilainen, Kerttu Majander, Thiseas C. Lamnidis, Luca Traverso, Johannes Krause, Antti Sajantila, Elina Salmela, Päivi Onkamo, Jussi-Pekka Taavitsainen

**Affiliations:** 1 Department of Biology, University of Turku, Turku, Finland; 2 Department of Archaeogenetics, Max Planck Institute for Evolutionary Anthropology, Leipzig, Germany; 3 Faculty of Biological and Environmental Sciences, University of Helsinki, Helsinki, Finland; 4 Department of Archaeology, University of Turku, Turku, Finland; 5 Museum Centre Vapriikki, Tampere, Finland; 6 Finnish Museum of Natural History, University of Helsinki, Helsinki, Finland; 7 Oulu Museum and Science Centre, Oulu, Finland; 8 Department of Environmental Sciences, University of Basel, Basel, Switzerland; 9 Department of Forensic Medicine, University of Helsinki, Helsinki, Finland; 10 Forensic Medicine Unit, Finnish Institute of Health and Welfare (THL), Helsinki, Finland

**Keywords:** Ancient DNA, Stable isotopes, Mobility, Dietary reconstruction

## Abstract

**Background:**

In northern Finland, the 17th–19th centuries CE marked a transition from the semi-nomadic lifestyle of the Sámi, the indigenous people of northern Fennoscandia, to agriculture brought by Finnish settlers. This transition led to the disappearance of the Kemi Sámi language and the assimilation of their speaker communities into the Finnish population. Inhumation burials predating the Finnish settlement are rare. The oldest known burial from the region, dating to the turn of the 16th–17th centuries, comes from Kitka, Kuusamo. This study investigates the genetic, isotopic, and cultural aspects of the Kitka burial.

**Results:**

The individual shows a clear genetic affinity with modern Sámi, and within Finland, shares the highest identity-by-descent (IBD) connectivity with present-day individuals living in the northeastern part of the modern administrative region of Lapland. Isotope data indicate that the individual spent their childhood farther north or northeast of their burial site, and reveal dietary changes associated with long-distance mobility over the course of their life. Strikingly, the results indicate that the individual resided outside of Finland, possibly in Iceland, during late childhood. The absence of a freshwater dietary signal in adulthood may suggest that the individual arrived in Kitka only shortly before their death.

**Conclusions:**

The Kitka individual likely had genetic roots in the areas around the northeastern border of Finland, but travelled far from this region during their lifetime. These findings provide insights into the life of a historical Sámi individual and illustrate how bioarchaeology can contribute to a more nuanced understanding of Sámi histories.

**Supplementary Information:**

The online version contains supplementary material available at 10.1186/s12864-026-12962-x.

## Background

Sámi are the indigenous people of northern Fennoscandia. They speak Sámi languages that belong to the Finno-Ugric language family. Genetically, they are clear outliers in the European genetic landscape [1–4] and carry a unique composition of uniparentally inherited markers [5–7] and a pronounced proportion of Siberian-like ancestry [4]. Notably, the Sámi’s Finno-Ugric-speaking geographic neighbours – Finns, Estonians and Karelians – also carry such Siberian-like ancestry, but in a much smaller proportion [4, 8].

The core Sámi region, Sápmi, covers the northernmost parts of modern Norway, Sweden, Finland and northwestern Russia. However, people carrying Sámi-like ancestry and/or speaking Sámi languages have historically inhabited a larger region, as evidenced by place names in southern Finland [9–11] and ancient DNA [8]. There is strong evidence of Sámi-related genetic admixture in non-Sámi Scandinavians [12–14], the Sámi-related ancestry being most prominent in northern Scandinavia and gradually decreasing southwards. This pattern of admixture has been present at least from the Viking period (750–1100 CE) onwards in modern-day Sweden and Norway [15], and it is likely that a similar pattern of admixture exists also in Finland [16].

In Finland, the southern border of Sámi inhabitation has retreated northwards as Finnish settlement spread to the north [17]. Until the 15th century CE, permanent Finnish agricultural communities were mostly situated in the southern and western regions of Finland, while northern inland areas that were less suitable for agriculture were inhabited by fishing and foraging groups who were at least partially Sámi-speaking [18]. During the 16th century, burgeoning population numbers, agrarian innovations and shifting geopolitical pressures propelled Finnish settlers northward. Although contemporary sources record marriages between Sámi and Finns [9, 19, 20], and attest that some Sámi individuals assimilated into agrarian communities and a few Finns were integrated into the Sámi communities, the overall Finnish influx displaced the Sámi who had been inhabiting regions south of present-day Sápmi. This led to the disappearance or diminishing of indigenous cultures from large swathes of the country. In light of these profound demographic shifts and the relative paucity of written records, reconstructing Sámi history stands to gain significantly from ancient DNA sequencing and complementary bioarchaeological approaches.

**Fig. 1:**
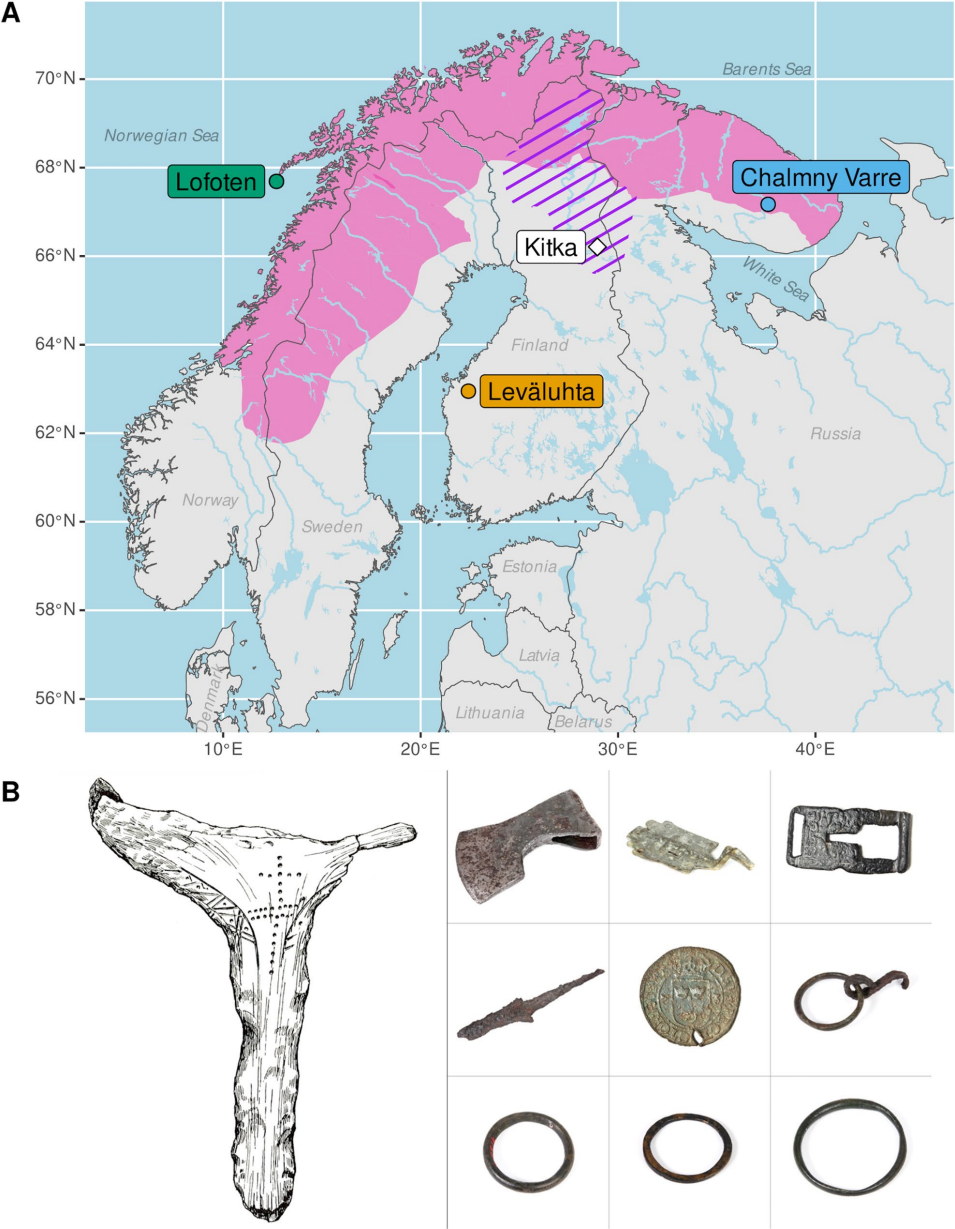
**A**. The individual’s burial place (white diamond), the current area of Sámi languages (pink) and the historical area of Kemi Lappmark (purple stripes). Coloured circles mark the burial locations of published ancient individuals with Sámi-related ancestry used for comparison in the DNA analyses. **B**. Artefacts discovered from the grave: a drum hammer made of a reindeer antler (left), an axe, a tin bird, a belt buckle, a knife, a silver coin (Swedish 2 öre of John III minted in 1573) carried as a pendant or amulet at the neck, and copper rings. Drawing: Kopisto 1971; Photos: Maisa Lukkari, Oulu Museum and Science Centre, Northern Ostrobothnia Museum collection

In 1970, a historical inhumation grave was discovered on the shore of lake Yli-Kitka in Kuusamo, a region known to have been inhabited by Sámi before the arrival of Finnish migrants in the 17th century [19] (Fig. 1A). The well-preserved skeleton belonged to a 40-year-old male [21], who had been buried at the turn of the 16th–17th centuries, based on the dating of a coin pendant found in the grave (Fig. 1B). The burial differed from typical Finnish inhumation burials of the time and contained objects associated with Sámi cultural heritage, particularly finds associated with a drum kit (Fig. 1B). Based on the drum kit and a bird pendant, the individual has been interpreted as a Sámi ritual specialist (noaidi in Northern Sámi). Noaidis were known to use drums (*goavddis* in Northern Sámi) when communicating with the spiritual realm [22, 23]. However, the presence of a drum kit in the grave does not definitely prove the individual’s role as a ritual specialist. Sources indicate that each Sámi dwelling had its own drum, and that even ordinary Sámi individuals could use them for divination and be buried with one [19, 24, 25].

Historically, the grave would have been located within the Kitka siida, one of the southernmost Sámi village communities [25]. These communities were kin-based social units that collectively managed subsistence activities, coordinated seasonal mobility, regulated resource use, and maintained territorial rights. The modern population of the Kuusamo region is Finnish-speaking, but during the time of the burial, the area was inhabited by a Sámi population speaking Kemi Sámi – now-extinct varieties of Sámi language resembling Eastern Sámi languages such as Inari Sámi and Skolt Sámi [26, 27]. The speakers of Kemi Sámi inhabited the drainage area of the Kemi River, which formed a major part of the historical Swedish administrative region of Kemi Lappmark. Although the Kemi Sámi culture does not belong to the living Sámi culture of today, it is an important part of the Sámi history in northern Fennoscandia.

The current inhabitants of Kuusamo descend mostly from a relatively small number of Finnish migrants and exhibit signs of strong genetic drift and long tracts of linkage disequilibrium, which have made them an attractive model population for identification of disease-related genes and variants [28, 29]. The Kitka individual is currently the only known archaeological individual from the Kuusamo region. We used DNA analysis to identify the individual’s phenotypic characteristics, population-level genetic background, and genetic connections to present-day Finns and Sámi. Stable isotope analyses of carbon, nitrogen, oxygen, and strontium were used to determine the individual’s places of residence, diet, and lifestyle at various stages of life. Although our study focuses on a single individual, it contributes to the broader understanding of the history of the Sámi people.

## Results

We extracted ancient DNA from the individual’s tooth sample (KUU001) and found it to contain 2% of endogenous human DNA (see Methods). We generated both double- and single-stranded DNA libraries that were enriched for 1.2 million genome-wide, ancestry-informative SNPs [30]. From the double- and single-stranded data, we obtained an on-target coverage of 0.2X (KUU001_ds) and 1.1X (KUU001_ss), corresponding to 220,800 and 509,496 SNPs, respectively. Contamination estimates for both the nuclear and mitochondrial genomes indicated < 5% of modern DNA contamination.

We also investigated genetically-inferred phenotypic traits to provide individual-level biological information and selected health-associated markers. Based on the relative coverage of the sex chromosomes (Data S1), the individual was genetically male, consistent with the osteological analyses. HIrisPlex analysis [31] indicated that the individual likely had fair skin, blue eyes and dark hair. We also screened the individual for seven genetic conditions belonging to the Finnish disease heritage that have higher prevalence in northern and northeastern Finland [32, 33] (Table S1), but no known disease-associated mutations were detected. The individual was a likely carrier of an HLA-B27 allele, which has been associated with several medical conditions, including rheumatic diseases such as juvenile rheumatoid arthritis and ankylosing spondylitis [34]. However, the examination of the individual’s bones found no evidence of degenerative joint disease [21]. The data were also screened for potential pathogen DNA with the HOPS pipeline [35], but no ancient pathogens were detected.

### The Kitka individual shared genetic ancestry with the present-day Sámi

The individual carried mitochondrial haplogroup V and Y-chromosomal haplogroup N1a1a1a1a2a1a1a1 (Z19813). Both are frequent in the present-day Sámi [7, 36]. The genome of the individual had several short and medium-length (4–20 cM) runs of homozygosity, a pattern consistent with small population size rather than recent inbreeding [37] (Fig. S1).

**Fig. 2:**
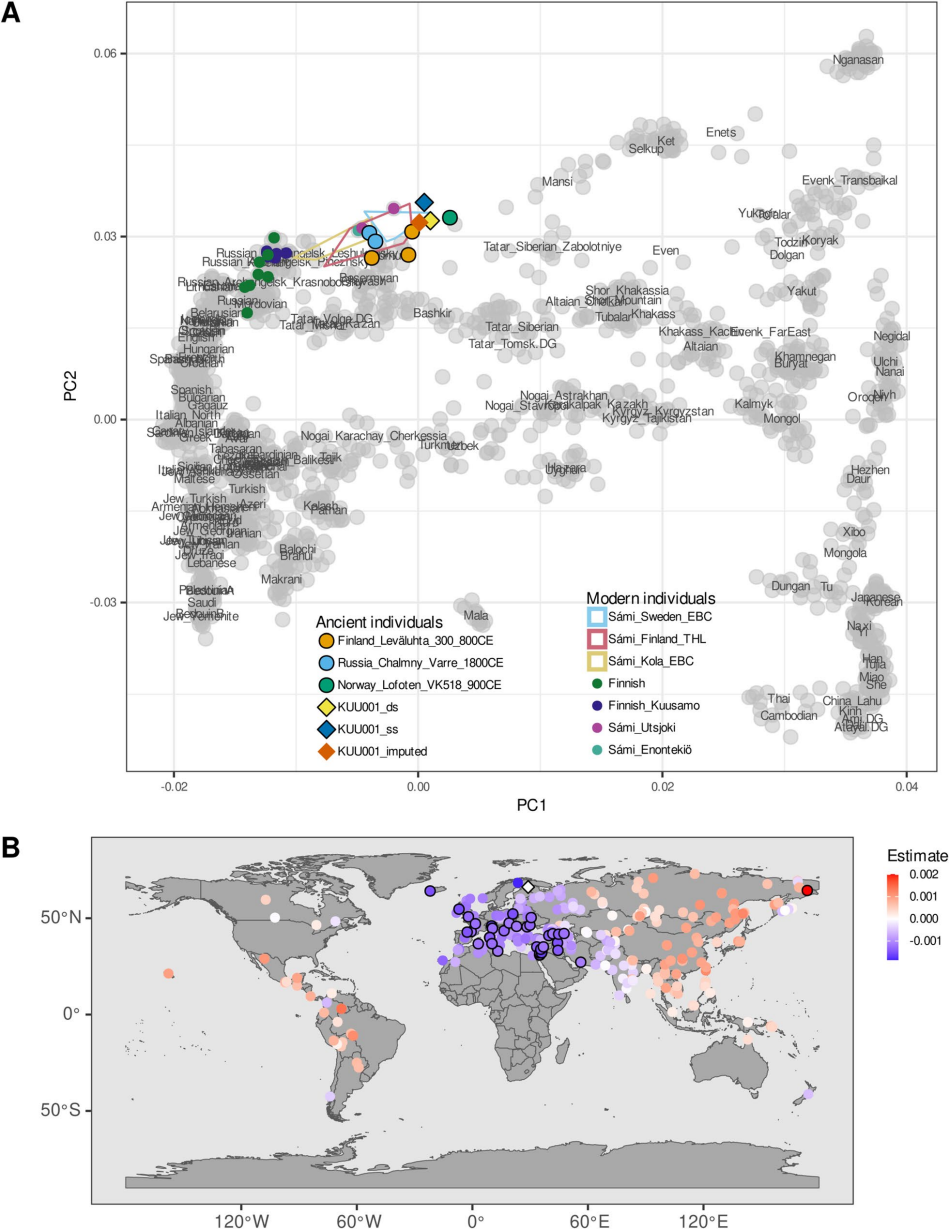
**A**. Principal component analysis (PCA). PC space is spanned using present-day individuals from 161 groups, including Finnish and Finnish_Kuusamo, and the imputed genome of the Kitka individual (KUU001_imputed; circles and a diamond without outlines). Labels show the median coordinates of each group. Ancient individuals (outlined circles and diamonds) and low-coverage present-day Sámi (convex hulls) are projected onto the PCs. CE: Common Era; EBC: Estonian Biocentre; THL: Finnish Institute for Health and Welfare. **B**. F4 estimates from the test of symmetrical relatedness between the Kitka individual and present-day Sámi from Utsjoki, Finland (*f*4(Mbuti.DG, Test; Saami.DG, KUU001_ss)). Outlined circles denote significantly non-zero (|Z| ≥ 3) estimates; blue shades denote extra affinity to the population in question in present-day Sámi, and red in the Kitka individual. A white diamond indicates the burial location of the Kitka individual

In principal component analysis (PCA), both pseudohaploid and imputed versions of the individual’s genome fall close to present-day Sámi and ancient individuals with Sámi-related ancestry: three individuals from the Iron Age Leväluhta (sometimes spelt Levänluhta; 300–800 CE) [8, 38] water burial in western Finland, and a Viking Age individual VK518 (900 CE) [39] from the Lofoten Islands in northern Norway (Fig. 2A); however, none of these individuals has a clear cultural connection to modern Sámi. The Kitka individual and VK518 both fall adjacent to a cline formed by present-day Sámi from Sweden, Finland, and the Kola Peninsula. Notably, this cline appears to oppose geography: the geographically easternmost Sámi from the Kola Peninsula fall closest to West Europeans, whereas the geographically westernmost Swedish Sámi fall furthest east on the “Siberian cline”; the Sámi from Finland fall in between the two. The affinity to the east correlates with the number of Sámi people in these three countries, suggesting that the reduction of eastern ancestry may be a product of admixture with the surrounding non-Sámi groups. The Kitka individual falls on the eastern edge of the Swedish Sámi group, which suggests that the individual may carry more Siberian-like ancestry than present-day Sámi from Finland do.

Consistent with PCA observations, outgroup F_3_ statistics with present-day populations show that the individual shares more drift with the present-day Sámi than with any other tested present-day group (Fig. S2A; Data S5; Methods). When compared to published ancient (Iron Age, medieval, and historical) individuals from northern Europe [8, 38–43], the Kitka individual shares most drift with VK518, Leväluhta, and historical Sámi from Chalmny Varre (Data S5). These three groups, the Kitka individual, and present-day Sámi show very similar ancestry composition in ADMIXTURE analysis across all Ks (Fig. S3).

We used F_4_ statistics to test whether the Kitka individual and present-day Sámi from Finland are symmetrically related to other present-day populations. The test *f*_*4*_(Mbuti.DG, Test; Saami.DG, KUU001_ss) gave significantly negative (Z ≤ -3) estimates for several West Eurasian populations, indicating that present-day Sámi carry more West Eurasian ancestry than the Kitka individual does (Fig. 2B). In the same test, the Kitka individual showed positive estimates of F_4_ relative to several East Asian and Siberian groups, although most of these estimates were not statistically significant.

When replacing Saami.DG with historical Sámi from Chalmny Varre, we obtained significantly positive (Z ≥ 3) estimates of F_4_ for many East Asian and Siberian groups, but no significantly negative estimates (Fig. S2B). Despite the apparent flip in significance, which is likely influenced by data type biases and small sample sizes, the directionality of F_4_ affinities remains the same in both comparisons. Overall, the results are consistent with the PCA analysis and indicate that the Kitka individual carried more Siberian-related ancestry than the present-day Sámi from Finland and the historical Sámi from the Kola Peninsula. Additionally, F_4_ statistics suggest that the present-day Sámi in Finland may have received gene flow from the surrounding populations with West European ancestry.

In contrast, a similar comparison with Leväluhta (*f*_*4*_(Mbuti.DG, Test; Finland_Levanluhta, KUU001_ss)) and VK518 (*f*_*4*_(Mbuti.DG, Test; VK518, KUU001_ss)) resulted in only one significant F_4_ estimate: the Kitka individual shares significantly more alleles with the present-day Sámi than Leväluhta does (Fig. S2B). VK518 and the Kitka individual, on the other hand, were symmetrically related to all tested present-day populations. This result may, however, reflect a lack of statistical power due to the relatively low genomic coverage of VK518 and the Leväluhta individuals.

To study if the Kitka individual shares a recent common ancestor with any of the published ancient individuals, we called IBD (identical-by-descent) segments between the Kitka individual and other ancient genomes using ancIBD [44]. We found one connection that passed our detection threshold (see Methods): the Kitka individual shared over 60 cM of IBD segments with VK518 from Viking Age Norway, and the longest shared segment between the pair was over 21 cM in chromosome 12 (Fig. S4; Data S8). This amount of IBD sharing appears incompatible with the temporal distance, approximately 800 years, between the Kitka individual and VK518, even under the small population sizes expected for historical Sámi. While the Kitka individual’s burial is securely dated based on the coin pendant placed in the grave, VK518 lacks a radiocarbon date and an unambiguously documented archaeological context: Margaryan et al. (2020) note that the burial assemblage was at least partially intermixed with another set of remains during curation and storage. In addition, VK518 shares distant but detectable IBD connections with early medieval individuals from southern Finland [45]. Taken together, these observations suggest misattribution of VK518 to a Viking Age context. Based on genetic data, the individual is more likely associated with historical Sámi groups in or close to Finland.

We did not detect an IBD connection between the Kitka individual and either of the two Chalmny Varre individuals. The individuals from Leväluhta could not be included in the ancIBD analysis due to their low coverage.

### IBD sharing connects the Kitka individual to northeastern Lapland

To explore the genetic affinities of the Kitka individual within Finland, we ran outgroup F_3_ statistics *f*_*3*_(KUU001_ss, Test; Mbuti.DG), where Test represented the present-day people from 115 Finnish municipalities where whole-genome sequencing data were available (‘THL_WGS’; see Methods and Supplementary Note), grouped into 27 regional analysis groups (Data S3). The individual shared most drift with present-day people from the administrative region of Lapland (Lappi) (Fig. S5A; Data S6).

The burial of the individual is located within the historical Kitka siida, which included northern parts of the present-day Kuusamo and Posio municipalities (Halinen 2025: 250). We tested if the present-day individuals from these municipalities share excess alleles with the Kitka individual compared to individuals from other Finnish regions by running F_4_ statistics in the form *f*_*4*_(Mbuti, KUU001_ss; Kitka, Test), where Kitka corresponds to present-day individuals from Kuusamo and Posio, and Test corresponds to 26 other regional analysis groups. However, no such relationship could be found: Only three regions from the western and southern coast of Finland shared significantly fewer alleles with the Kitka individual than the present-day inhabitants of the Kitka region do. (Fig. S5B; Data S6), suggesting that gene flow from the Kitka individual, or the population the individual belonged to, was not exclusive to the Kitka region.

**Fig. 3:**
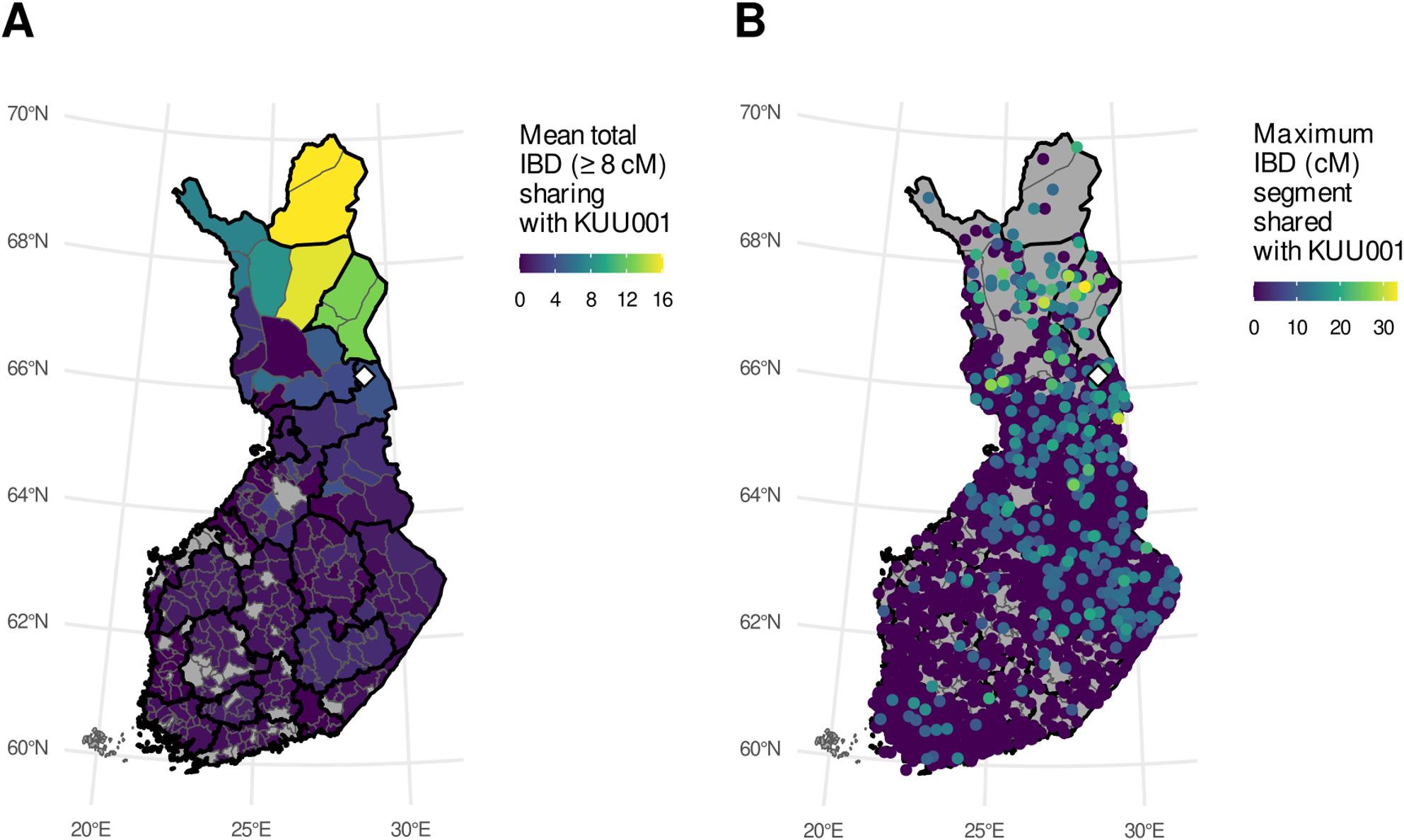
**A**. IBD sharing of Kitka with the present-day population of Finland. Mean sums of IBD segment lengths (≥ 8 cm) shared between the Kitka individual (KUU001) and present-day inhabitants of Finland, averaged over regional analysis groups. Black outlines highlight regional analysis groups that comprise more than one municipality. Grey polygons show municipalities that have no data. A white diamond marks the burial place of the Kitka individual. **B**. The longest IBD segment shared between the Kitka individual and each individual in the Finnish dataset is indicated by the colour of the circles. Individuals have been assigned a random coordinate within the area of their regional analysis group

F statistics measure genome-wide allele sharing and are potentially insensitive to genetic contribution from one or a few ancestors, even if they came from a genetically distinct population. Therefore, we examined IBD sharing to detect individual-level genetic connections between the Kitka individual and present-day individuals from Finland. For IBD analyses, we used a dataset consisting of genotyping array data of 3499 present-day individuals from 246 Finnish municipalities (‘THL_HCE’; see Methods and Supplementary Note), grouped into 97 regional analysis groups. We calculated the total length of IBD segments shared between the Kitka individual and each modern individual and averaged those lengths over the regional analysis groups. We find the highest mean IBD sharing with the Kitka individual in northern Lapland (Inari and Utsjoki), Sodankylä, and eastern Lapland (Salla, Savukoski and Pelkosenniemi) (Fig. 3A; Data S7). The Kitka region, although located immediately adjacent to the highest-sharing regions, shows much lower levels of IBD sharing with the Kitka individual.

Since IBD segments are broken down by recombination in every meiosis, longer segment length indicates, on average, shorter genealogical distance separating the two individuals from their common ancestor. The median length of inferred segments was 12.3 cM, which is consistent with the temporal distance between the Kitka individual and the present-day Finns, but we discovered segments ≥ 20 cM in 28 individuals (Fig. 3B; Data S7). Three individuals from eastern Lapland, Sodankylä, and Kuusamo carried segments shared with the Kitka individual that were over 30 cM. Notably, we did not detect long segments in northern Lapland (Inari and Utsjoki), although the mean sum of IBD segment lengths in these regions is similar to eastern Lapland and Sodankylä. However, the chances of detecting long IBD segments are likely affected by large differences in sample sizes in the northern municipalities (see Data S4).

We further calculated the fraction of individuals sharing IBD segments with the Kitka individual for every regional analysis group. We found that 9.1% of the studied modern individuals across Finland shared at least one IBD segment of length ≥ 8 cM with the Kitka individual, while in Kuusamo and Posio, the sharing was 25.2% and 31.6%, respectively (Fig. S6A, Data S7). However, the highest fraction of individuals sharing IBD segments with the Kitka individual, 66.7%, was in northern Lapland (Fig. S6A).

IBD sharing with the Kitka individual in the Finnish population shows a general trend of north-to-south gradient, which is consistent with the historically known fact that Finns have mixed with the Sámi in the northern regions (e.g [9, 17]). Thus, the observed IBD sharing pattern may simply reflect the level of Sámi-related admixture in each region. To explore this, we replicated the IBD analysis but substituted the Kitka individual with two present-day Sámi genomes. We found that the fraction of present-day Finns sharing segments with either of the two modern Sámi was 8.1%, and the general pattern showed a visually similar geographic gradient in IBD sharing with Sámi as seen for the Kitka individual (Fig. S6B). To test this, and to account for the many regions with no IBD sharing, we fit a zero-inflated hurdle model with beta regression to model the distribution of the Kitka individual-related segments as a function of Sámi-related segments and latitude (Supplementary Notes and Fig. S6C). The residuals of the model reveal a geographically stratified pattern: IBD sharing with the Kitka individual is stronger than predicted in eastern Lapland and, to some extent, in central Lapland, North Ostrobothnia, and northern Kainuu. Conversely, the people living in western Lapland and most of southern Finland share fewer IBD segments with the Kitka individual than they do with present-day Sámi. Thus, the IBD connectivity to the Kitka individual in eastern Lapland is not fully explained by the patterns of modern Sámi-related admixture.

### Stable isotopes reveal marine diet and high lifetime mobility

To study the Kitka individual’s lifetime mobility and diet, two teeth (a premolar and third molar) and a rib were sampled for isotope analyses of carbon (ẟ^13^C), nitrogen (ẟ^15^N), oxygen (ẟ^13^C) and strontium (^87^Sr/^86^Sr) (see Table 1). Collagen for the analysis of ẟ^13^C and ẟ^15^N and Ag_3_PO_4_ for ẟ^18^O were successfully extracted from the targeted samples. All extracts met the established quality criteria for good collagen preservation [46–48]. The results of the isotopic analyses are reported in Table 1. The carbon and nitrogen isotope values indicated a dietary change from childhood (premolar) to adolescence/adulthood (third molar), with ẟ^13^C values increasing from − 19.6‰ to an average of -18.5‰, and a parallel decrease of ẟ^15^N values from 15.3‰ to an average of 13.8‰. Likewise, there was a distinct difference between childhood and adolescent strontium isotope values, decreasing from 0.72926 to a low value of 0.70813. The rib ^87^Sr/^86^Sr value of 0.73976 suggested a generally high strontium isotope value for the soil of the burial environment. The ẟ^18^O values of the different tissues showed a mean of 11.4 (± 0.59)‰, which corresponds to a ẟ^18^O value of drinking waters roughly from − 17 to -15‰ [49].

**Fig. 4:**
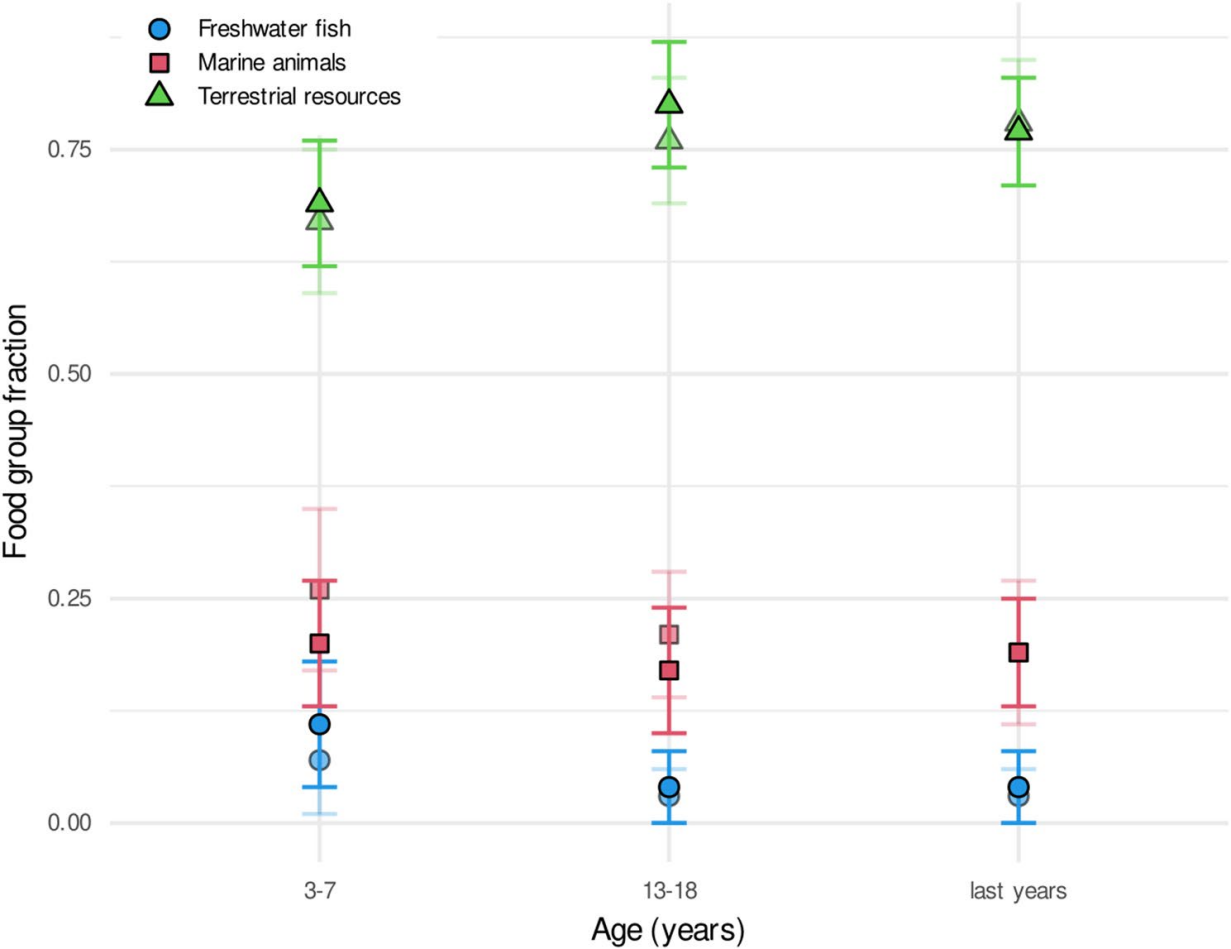
Modelled food group fractions α_TR, FF, MA_ for the Kitka individual’s samples according to the corresponding living years. The dark and light symbols correspond to the assumption of the origin of the marine food as the Barents and White Seas, respectively

Dietary modelling on teeth and rib collagen ẟ^13^C and ẟ^15^N data provides estimates for food group intakes given in Table S2 and illustrated in Fig. 4. We obtained nearly the same relative contributions of food groups (terrestrial resources TR, freshwater fish FF and marine animals MA) regardless of the assumption of the origin (Barents Sea or White Sea) of marine carbon. Terrestrial sources (αTR = 67–80%) explain most of the dietary input. It is accompanied by significant marine dietary contributions (αMA = 17–26%). It is noteworthy that freshwater dietary input appears nearly negligible for the years of adolescence/adulthood, contrasting the childhood diet with a higher freshwater component (see also Fig. S7). This holds regardless of the origin of marine carbon. Overall, the diet seems to be strongly terrestrial with a fairly large aquatic component, thus slightly elevating the protein content.

**Table 1:** Stable isotope values

Sample	Tissue	Yield%	C%	*N*%	C/*N*	ẟ^13^C, VPDB	ẟ^15^*N*, AIR	ẟ^18^O, VSMOW	^87^Sr/^86^Sr	Years
FDI35	enamel	-	-	-	-	-	-	12.2	0.729262	3-4.5
	dentine	n/a*	38.2	14.2	3.1	-19.6	15.3	10.9	-	3–7
FDI48	enamel	-	-	-	-	-	-	-	0.708134	9–13
	dentine	16.2	42.0	14.9	3.3	-18.4	13.9	10.9	-	13–18
rib	bone	12.7	44.4	16.2	3.2	-18.5	13.7	11.6	0.739766	last year/ Sr = diagenetic^‡^

* collagen yield not available due to vial breakage

‡ ^87^Sr/^86^Sr value of the rib bone represents the isotopic composition of Sr in the burial soil

As a sensitivity analysis, we tested numerous food group compositions during modelling by altering both isotopic baselines and corresponding macronutrient concentrations of foods (Tables S3, S4). Since the FF component is small for adolescence/adulthood, we assumed a scenario of the diet containing terrestrial plants (TP), terrestrial animal resources (TAR) and marine animals (MA) by neglecting FF and dividing the TR food group into TP and TAR. However, this resulted in extremely wide and indistinguishable distributions of TP & TAR food group fractions (Fig. S8) due to the close similarity of their isotopic baselines (Table S3), while the MA fractions consistently remained close to 20%. Therefore, the selection of the TR + FF + MA scheme seems reasonable. In addition, when assuming TR consisting of 80% of reindeer meat and 20% plants (i.e. TRreindeer + FF + MA, tested for third molar FDI48, i.e. living years of 13–18), the protein fraction increased to 69%. This seems unrealistically high, as this would potentially lead to so-called rabbit starvation syndrome, which can cause fatal health effects [50]. Therefore, it seems that the terrestrial diet was more versatile. In summary, the modelling suggests a strong reliance on both terrestrial and marine food, and considerable freshwater food consumption during childhood.

The ẟ^18^O and ^87^Sr/^86^Sr values of skeletal bioapatite reflect the values of drinking water and ingested foods, making them valuable tracers for the geographical place of residence at the time of tissue formation. The use of oxygen isotopes in provenancing individuals is based on the predictable, climate-related geospatial pattern of ẟ^18^O values in global precipitation, and consequently in drinking waters, where values decrease towards increasing latitude and altitude, as well as continental interiors [51]. In Finland, the highest ẟ^18^O values (ca. -11.5 per/mil) of precipitation are found in the southwest and the lowest (ca. -15 per/mil) in the far north [52]. Factoring in the cold climate anomaly (Little Ice Age; e.g. [64]). coinciding with the individual’s lifetime and likely to have lowered the ẟ^18^O values of precipitation, the very low ẟ^18^O values obtained from the individual’s teeth are consistent with a place of residence in the high north during both early childhood and adolescence/early adulthood.

Skeletal strontium tracks the isotopic values of bioavailable strontium in the soils, derived from the bedrock of the geographical area where food is sourced. The bedrock of the Yli-Kitka region, Paleoproterozoic (2.2–2.06 Ga) sericite quartzites, mafic volcanites and gabbros, can be expected to show both high and highly variable ^87^Sr/^86^Sr ratios (Q1–Q3: ca. 0.72 to 0.83; median 0.742) [53]. The rib bone ^87^Sr/^86^Sr ratio at 0.7397, reflecting the bioavailable Sr of the burial soil, is in agreement. The dental ^87^Sr/^86^Sr values of the Kitka individual indicate a change in the place of residence and consequently, food Sr catchment, between early (ca. 3–7 years; premolar) and late childhood (ca. 9–13 years; third molar). The higher value obtained from the premolar, 0.7292, is consistent with terrestrial sources of Sr from the old Precambrian bedrock of the Fennoscandian shield area in general (e.g [53, 54]), and compares with the ^87^Sr/^86^Sr values of the rivers Iijoki and Kemijoki (0.730 and 0.734, respectively) [54] draining the adjacent Archaean and Proterozoic basements of northern Finland. In stark contrast, the third molar yielded a ^87^Sr/^86^Sr value at 0.7081, the lowest value measured in Finnish biological modern and archaeological samples so far. The value is lower than the ^87^Sr/^86^Sr levels of global sea water (ca. 0.7092), and without an additional food source with exceptionally low ^87^Sr/^86^Sr values well below 0.708, not currently known to exist in Finland, is unobtainable even on a heavily marine-based diet. The value suggests a place of residence outside the present-day Finnish borders at the time of tooth crown formation.

## Discussion

The archaeological context of the Kitka individual’s grave indicates a connection to indigenous Sámi heritage. The grave goods include a drum hammer (*ballin* in Northern Sámi) decorated with a motif often interpreted as an Orthodox cross [22], which has been linked in earlier interpretations to the integration of Christian symbols into indigenous ritual contexts and possible contacts with Orthodox Skolt Sámi in the Kola Peninsula [23, 55]. However, the motif is too indeterminate for a definitive interpretation as an Orthodox cross, as its proportions are incorrect and deviate from the standard form in both bar length and orientation (Fig. 1B). Also, genetic connection to the historical Sámi individuals from Chalmny Varre in the Kola Peninsula indicated shared ancestry only in a broad sense: no IBD connection was detected between the Kitka individual and the two analysed Chalmny Varre individuals. However, Chalmny Varre is located in the eastern part of the Kola Peninsula, whereas Skolts inhabit its western parts [56]. Overall, the individual’s material culture indicates broad cultural exchanges and shared practices across northern Fennoscandia amidst the territorial and tax disputes between Sweden, Russia, and Norway.

The individual was buried in an area historically inhabited by the indigenous Kemi Sámi, a population whose culture and language were replaced by the customs of Finnish settlers shortly after the individual’s lifetime. This burial thus presents a rare opportunity to explore the Sámi history of the region on an individual level.

The individual’s genetic ancestry aligns closely with present-day Sámi. The genome-wide analyses show high allele sharing with both contemporary and historical Sámi. The individual’s mitochondrial haplogroup V is one of the two predominant haplogroups among today’s Sámi, alongside U5b1b. Similarly, the Y-chromosomal haplogroup falls within the branch constituting nearly half of the Sámi paternal lineages, though it appears at an even higher frequency among Finns. Levels of ROH in the individual’s genome suggest an origin in a small population, which aligns with known population sizes for historical Sámi groups and their generally low population densities across Fennoscandia [18, 57].

The Kitka individual’s grave has gained considerable media attention in Finland in recent years (e.g [58–60]), with public debates centred on where the remains should be located and what should be done with them. Some Kuusamo municipal council members have controversially called for a DNA analysis to “determine where the deceased belongs” [59, 61]. This view exemplifies the popular but false notion that genetics could conclusively define origins, ethnicity, and cultural identity (see [62–64]. Ethnicity is inherently social and based on personal identities and social relations, not genetic markers [65]. The Kitka individual’s grave goods indicate a strong Sámi affiliation, but the story that emerges from the combination of DNA and isotope analyses is more nuanced than a straightforward interpretation based only on grave goods or any of the methods alone.

### Diet and lifetime mobility of the Kitka individual

The Sámi have historically maintained semi-nomadic lifestyles, often based on fishing, with reindeer herding emerging in northernmost Fennoscandia between the 14th and 17th centuries [66]. In Kemi Lappmark, the Sámi subsistence economy centred on freshwater fish during spring through autumn, and hunting in winter [9, 67], while reindeer herding was never adopted. Stable isotope modelling of the Kitka individual’s remains shows significant reliance on terrestrial and marine food throughout life, but it also suggests the individual consumed freshwater resources in childhood. Thus, the individual was likely born in an area where both freshwater and marine resources were available. Given that freshwater sources were crucial in Kemi Sámi diets [67], the lack of freshwater signal in the individual’s late childhood diet suggests adolescence and adulthood somewhere else than in Kuusamo, and rather in a more northerly region near seashores. The ẟ^13^C and ẟ^15^N baselines of the Barents and White Seas (or Bothnian Bay, see Methods) provide nearly similar modelled food group fractions (Fig. 4), thus preventing further judgment on specific origins based on marine dietary sources alone. Further, as isotopic signatures of the Barents and Norwegian Seas are also similar [68], a broader origin of marine influence may also be possible. Nevertheless, a proximity to the seashore and/or strong reliance on anadromous fish may provide fundamental elements behind such a significant dietary component.

Comparative dietary isotope values of C and N of historical Sámi exist from Silbojokk in inland Sweden, Rounala in northern Sweden, and Kirkegårdsøya and Gullholmen in northern Norway [69–71]. Individuals buried in Kirkegårdsøya had a homogenous and extensively marine diet, whereas both Rounala and Gullholmen individuals showed higher dietary variation with a generally moderate marine component and potentially diverse geographic and cultural origins of the individuals. Likewise, inhabitants of Silbojokk were likely multi-cultural, which is reflected in the variation in the food culture. The Kitka individual’s dietary isotope values representing their early childhood do not correspond to values from any of these comparative sites, suggesting a different dietary composition with a considerable fraction of freshwater fish. In contrast, the values measured from FDI48 and rib (later years) fall within the large range of values measured from Gullholmen. In this respect, the Kitka individual’s dietary profile eventually aligns with patterns associated with mobile and regionally connected coastal Sámi communities.

Oxygen and strontium isotopes provide further insights into the individual’s lifetime mobility. The early childhood ^87^Sr/^86^Sr value aligns with the results from oxygen isotopes, which are consistent with origins in northeastern Finland. However, the conspicuously low ^87^Sr/^86^Sr ratios (below 0.7085) in the third molar are anomalous for the Precambrian crystalline bedrock of Finland, resulting in generally high ^87^Sr/^86^Sr values (> 0.720) of terrestrial bioavailable baseline values [38, 72–76]. We note, however, that a recently published model of bioavailable ^87^Sr/^86^Sr values over Fennoscandia [77] implies that ^87^Sr/^86^Sr values < 0.710 are prevalent in the Kuusamo area, although this starkly contradicts the currently available empirical indications of ^87^Sr/^86^Sr ratios in this region – the ^87^Sr/^86^Sr values obtained from the Kitka individual’s rib bone and river Iijoki [54]. This implies that the model by Armaroli et al. [77] might benefit from further validation in this particular area. The low ^87^Sr/^86^Sr value more likely suggests residence on young carbonate or volcanic terrains when the tooth crown mineralised. Sámi communities have had contacts with southern Norwegian coastal regions, especially Bergen [78, 79], which leads to speculation whether the individual had travelled there. However, human and animal ^87^Sr/^86^Sr values below 0.709 are uncommon in these regions, where such values have been considered to indicate migration from outside of Norway [80]. Comparable low values in humans are documented, for example, in northwestern Denmark [81], and certain parts of the UK and Ireland [82, 83], yet they are the most prevalent in Iceland, where values between 0.706 and 0.709 are common among individuals consuming local resources [84, 85]. Low ^87^Sr/^86^Sr values may also occur in parts of Siberia, where there are Phanerozoic sedimentary rocks that have been modelled to display ^87^Sr/^86^Sr ratios from 0.708 to 0.709 [86]. However, the lack of bioavailable baseline data from these regions limits a more precise evaluation of this possibility. The validity of the hypothesis of relocation to any of these regions cannot be reflected against ẟ^13^C, ẟ^15^N or ẟ^18^O values expected for typical diets or drinking water in these regions, as none of the other analysed isotope proxies reflect the same time period of formation. The formation of the root dentine tissues used for the analysis of C, N and O stable isotopes either pre- (3–7 yrs age) or post-date (ca. 13–18 yrs age) the crown enamel (ca. 9–13 yrs age) showing the anomalously low ^87^Sr/^86^Sr value.

If Iceland is the most suitable location according to strontium, is it possible that the Kitka individual spent time there during their youth? Sámi mobility has traditionally been viewed as locally confined and structured around seasonal hunting or reindeer herding transhumance. However, the idea of an area closed from outside influences has recently been replaced by a perception of a heterogeneous, outward-looking society [87–89]. The isotope evidence from the Kitka individual reflects not seasonal movements but longer-term relocations, suggesting that the individual lived outside the local area for extended periods. These findings align with research that emphasises the historical connections of northern Fennoscandia. From the 13th century onward, Icelandic merchants operated along the Finnmark coast, and by the mid-16th century, English and Dutch fur traders were also active in the region [19, 66]. A distinctive house type (mangeromstufter) found in Finnmark and dated to ca. 1300–1550 CE has been linked to Sámi-Icelandic maritime trade [79, 90, 91].

Given the individual’s age at death (around 40) and the dating of the burial to the turn of the 17th century, a potential stay in Iceland would have occurred in the 1570–1580 s. This period coincides with the 25 Years’ War (1570–1595), during which Russians and Finns launched repeated military expeditions into each other’s territories [92]. Sámi communities in the northern borderlands were caught in the conflict and reportedly clashed with both sides [19]. One explanation for the individual’s journeys is that their youth was spent as a refugee on the Arctic coasts, possibly even reaching Iceland. By the late 16th century, several Sámi communities resided in northern Karelia near the White Sea [93]. Russian tax records report the capture of 33 Sámi families by Swedes in this area in 1589, with survivors fleeing the violence [93, 94]. Tax records from Kuusamo also note Sámi families evading taxation between 1579 and 1594 [19]. The Kitka individual’s grave goods reflect both eastern and western connections, while the isotope signature may indicate overseas mobility.

Kopisto [22] proposed that the pewter bird ornament found in the grave belonged to a Sámi noaidi costume, an interpretation repeated in later literature (e.g. [21, 23, 95]). While metal bird motifs can be connected to Sámi drums, there is no evidence for their attachment to clothing, and there is no archaeological or textual evidence that Sámi noaidis wore distinct shamanic attire like their Siberian counterparts. Moreover, the absence of a freshwater dietary signal during the final years of life suggests a late arrival in Kitka, possibly shortly before death. This supports an alternative interpretation: that the individual was merely passing through the region at the time of death. The findings also challenge earlier assumptions that the individual was a noaidi. Ethnohistorical sources describe the training of noaidis as beginning in childhood, and the role’s deep integration within a specific siida [95, 96]. The individual’s late arrival in Kitka raises doubts about whether the individual could have completed the required long-term training and had time to fully integrate into the social and spiritual structures of the community. Oral traditions do note that noaidi status could also arise after surviving three prolonged illnesses [25]. Osteological analysis indicates that the individual performed physically demanding labour during life [21]. Given that the noaidi’s role primarily involved spiritual and ritual functions, the physical profile could suggest a different social position. Altogether, the combination of high mobility, isotope evidence, osteological profile, and historical data calls for broader and more flexible interpretations of Sámi identities, movement, and social roles in the 16th-century north.

### Genetic legacy of the historical Sámi in the Finnish population

Sámi have admixed with their neighbouring populations, especially in the northern parts of Fennoscandia, which is evident in marriage records and has been demonstrated from genetic data for Sweden and Norway [12, 15]. Assuming that the Finns admixed with the local Sámi groups, we can use IBD sharing to locate the most likely provenance of the population that the Kitka individual belonged to. We cannot exclude the possibility that the individual is a direct ancestor of some present-day people in our data set, but it is not possible to recover the exact genealogy from such a distant relationship. Moreover, the lack of shared IBD segments cannot exclude a genealogical connection between two individuals when they are separated by several generations. Instead, these genetic connections should be seen as evidence of population contact between the ancestors of the present-day individuals and the population to which the Kitka individual belonged.

The peak of IBD sharing between the Kitka individual and present-day Finns matches relatively well with the northern part of the presumed extent of the Kemi Sámi speaker area before Finnish colonisation, supporting the assumption that the individual was genetically part of the extinct Sámi groups of this region. The southernmost parts of this area, which included the Kitka siida where the individual was buried, have much weaker IBD connections to the individual. While this observation may indicate that the individual’s population lived north of Kitka, it could also simply reflect the time or intensity of the Finnish colonisation. In the Kitka region, Sámi communities disappeared by the end of the 1600s, while in the northern regions they survived longer [25, 27].

Taken together, our IBD results indicate that the individual had a genetic origin relatively close, within 250 km, to the place where he was eventually buried. Such an origin is consistent with the Sr and O isotopes measured from the individual’s premolar and the observation of a freshwater dietary component during childhood. The distance falls approximately within, or slightly exceeds, the known mobility range of historical Sámi groups in the Kuusamo region [66]. The relatively strong marine dietary component during childhood is, however, somewhat puzzling as it possibly indicates a farther distance from the core Kitka region. Our data provides information on the IBD connections to the individual only within Finland, although the areas inhabited by historical Sámi groups did not follow the current geopolitical borders. However, similar, dense Biobank data from Russia, Sweden, and Norway either do not exist or were not available for this study. Thus, a potential genetic connection to areas outside of Finland could not be fully explored. Based on the combination of results from genetic and isotopic analyses, it can be speculated that the individual’s early childhood may have been spent near Kandalaksha Gulf in the northwestern corner of the White Sea, only 100 km from Finland’s eastern border, where both marine and freshwater resources would have been available. Alternative explanations for the marine dietary component during childhood could be consumption of migratory fish or traded stockfish from the White Sea. As the isotopic baselines of C and N of Bothnian Bay and White Sea resemble each other, dietary contribution from Bothnian Bay can not be excluded, but overall, the results better support northeastern residence.

The Finns have been extensively studied due to their history of relative isolation, genetic drift and unique disease heritage that still affect the modern population. Meanwhile, the role and influence of the Sámi and the likely long history of admixture between the Finnish and Sámi populations have often been overlooked. In our analysis, almost 10% of the studied modern individuals from Finland shared IBD segments with the Kitka individual, suggesting that the IBD pattern observed in this study is at least partially driven by Sámi-related admixture into the Finnish population. Indeed, we found similar levels of IBD sharing between the present-day Finns and the present-day Sámi. We note that this level of IBD sharing may be an underestimate due to the low SNP density of the modern Finnish reference panel. However, the spatial distributions of the Kitka individual-related and modern Sámi-related segments in the Finnish population have unique features, notably within northern Finland, suggesting that present-day Finns might retain remnants of the historical substructure of the Sámi groups who inhabited the area of modern Finland before the Finns. The results of this study highlight the need for a more comprehensive narrative, which will take into account the historical interactions between the Finnish and Sámi communities. Simultaneously, our results emphasise the complexities of constructing indigenous or other contemporary identities around genetics, and caution against employing genetic arguments to define Sámi or Finnish identities in present-day Finland.

## Conclusions

The Kitka burial offers a rare individual-level perspective on Sámi history at a time of cultural, political and demographic shift in northeastern Finland. The archaeological context links the individual to the Sámi cultural heritage. Similarly, genome-wide data demonstrate that the individual shared ancestry with both present-day and historical Sámi, as well as earlier ancient individuals from Fennoscandia. The population that the Kitka individual belonged to contributed most genetic ancestry to present-day Finns living in northeastern Finland. Moreover, almost one in ten of the present-day Finns shared IBD segments with the Kitka individual or present-day Sámi, underlining the role of Sámi–Finnish admixture in shaping the Finnish gene pool. These findings highlight the long-term genetic entanglement of these groups.

Isotopic evidence reveals a highly mobile life history and possible residence in regions as distant as Iceland, although the lack of empirical strontium baseline data prevents us from ruling out other possible directions. Such mobility demonstrates the extent of Sámi participation in cross-regional interactions, trade, and possibly increased movements during periods of conflict. Contrary to previous assumptions that the Kitka individual represents a historical Sámi local to the Kuusamo region, the lack of a freshwater dietary signal after childhood could indicate that the individual arrived at Kuusamo only shortly before death.

Together, the archaeological, genetic and isotopic data paint a picture of an individual whose life intersected with multiple cultural and geographic spheres. The results underscore the importance of integrating bioarchaeology with historical and ethnographic perspectives to understand Sámi pasts. They also caution against genetic determinism: while genetic data can connect past and present populations, ethnicity and identity remain shaped by social context rather than biological descent. 

## Methods

### DNA extraction, library production and sequencing

Laboratory work was carried out in the ancient-DNA laboratory of the Max Planck Institute for the Science of Human History in Jena (MPI-SHH) and sequencing in the facilities of the Max Planck Institute for Evolutionary Anthropology in Leipzig (MPI-EVA), Germany. We drilled approximately 50 mg of bone powder from the inner chamber of the dental pulp [97]. DNA from the bone powder was extracted following an optimised protocol for ancient DNA recovery [98, 99].

We used 25 µl of extract to build a double-stranded and UDG-half-treated DNA library [100–102]. The extract was treated with the USER enzyme to repair the DNA damage in the inner parts of the DNA molecules while retaining the damage at the terminal bases of the fragments.

The rest of the extract was converted into three single-stranded libraries that were not treated with the USER enzyme, following an automated single-stranded library preparation protocol [103, 104]. Each library was prepared from 30 µl of extract. All libraries were sequenced up to 5 million reads on the Illumina HiSeq 4000 System to obtain an overview of the DNA preservation in the sample. Libraries were then enriched for 1,237,207 genome-wide single-nucleotide polymorphisms using an in-solution target enrichment protocol [30, 105, 106]. The captured libraries were sequenced on a HiSeq 4000 platform with 75 single-end cycles, to approximately 40 million reads (for the double-stranded library) and 20 million reads (for single-stranded libraries).

### Bioinformatic processing and quality control

Sequenced reads were processed in the core automated pipeline at the MPI-EVA. Adapters were removed with leeHom [107] v1.1.5-ba378b6 using parameter --ancientdna. Reads were aligned to the human reference genome hs37d5 with BWA aln [108] version 0.7.12 with parameters adjusted for ancient DNA (-n 0.01 -o 2 -l 16500). We used nf-core/eager [109] v.2.4.5 pipeline for quality control, genotype calling, genetic sex determination and X chromosomal contamination estimation. DNA authenticity was confirmed based on patterns of DNA damage at the terminal positions of the sequenced molecules using DamageProfiler v0.4.9 [110]. Pseudohaploid genotypes were called with pileupcaller from sequenceTools (https://github.com/stschiff/sequenceTools), separately for double and single-stranded libraries to avoid biases arising from different UDG treatment. Prior to genotyping, two terminal bases from each end of the reads from the double-stranded libraries were trimmed with bamUtils [111] to remove remaining DNA damage. Single-stranded data was genotyped with the option --singleStrandMode. We calculated autosomal contamination using ANGSD [112] and mitochondrial contamination using Schmutzi [113]. Mitochondrial haplogroup was called from the endogenous consensus sequence provided by Schmutzi using Haplogrep 2 [114]. The paternal Y haplogroup was determined with Y-LineageTracker [115] using ISOGG 2019 positions and confirmed the haplotype call using PathPhynder [116]. We used hapROH to detect runs of homozygosity from the data [37].

### Reference datasets

Genotype data were merged with published, openly available modern and ancient genotype data using the Poseidon framework [117] and the data retrieved from the Poseidon Community Archive (https://www.poseidon-adna.org/#/). See Data S1 for package versions used in analyses. In addition, we included genotyping array data from present-day Sámi from the Kola peninsula and Sweden from Tambets et al. [4], and restricted data from various modern populations from Lazaridis et al. [3, 118].

In addition to publicly available genomes, we utilised whole-genome sequencing and genotyping array data from the Finnish Institute for Health and Welfare (THL). These data originated from the National Finrisk Study cohorts collected in 1992–2012 and the Health 2000 cohort (see Supplementary Notes for details). The data are available for researchers through an application procedure at https://thl.fi/en/research-and-development/thl-biobank. We restricted our analyses to those individuals whose parents were born in the same municipality. Furthermore, we excluded municipalities that were no longer independent municipalities in 2022 due to consolidation or other administrative changes.

### Genome-wide data analyses

We used principal component analysis (PCA) and unsupervised ADMIXTURE v1.3.0 [119] to visualise the genetic ancestry of the Kitka individual. PCA was done using smartpca [120] with parameters lsqproject: YES, popsizelimit: 25 and newshrink: YES. Principal components were calculated based on the genetic variation of 160 present-day Eurasian groups and the imputed genome of the Kitka individual (Supplementary Notes and Data S2). Pseudohaploid ancient genomes, as well as the genotyped present-day Sámi, were projected on those PCA coordinates.

To run ADMIXTURE, the data were first pruned using PLINK [121] v1.9 to exclude SNPs that had a minor allele frequency < 0.01. We also performed LD pruning using indep-pairwise with a window size of 200, a step size of 5 and an R2 threshold of 0.5. After pruning, our input data had 220,943 variants and 841 individuals representing 68 modern and ancient groups, including KUU001 (Supplementary Notes and Data S2). We ran five replicates of each K in the range of 2 to 16.

All F_3_ and F_4_ statistics were calculated using the xerxes v1.0.0.2 fstats tool from the Poseidon framework [117](https://poseidon-framework.github.io/#/). We used Mbuti.DG (*n* = 4) [122] as an outgroup in all calculations. Outgroup F_3_ statistics were calculated using the function F3vanilla.

For IBD and phenotype analyses, we pooled all the data produced from the sample KUU001 and imputed diploid genotypes using GLIMPSE [123]. Genotype likelihoods were first estimated with ATLAS, which takes ancient DNA damage into account [doi: 10.1101/105346].

IBD segments between the Kitka individual and published ancient genomes were called with ancIBD. We included published data, imputed with GLIMPSE as above, from the four papers that covered temporally proximate time periods and geographically relevant regions [8, 15, 39, 45]. We included only samples that had post-imputation posterior genotype probability > 0.99 in over 50% of the 1240k sites. Only 1240k sites were used in the ancIBD analysis, but sites were not further filtered for posterior probability. Furthermore, due to the limited quality of the individual’s imputed genome, we considered only those pairs that shared at least two segments of ≥ 10 cM in IBD.

IBD segments between the Kitka individual and present-day Finns were called using IBIS [124]. Imputed genotypes were filtered to only include positions with posterior genotype probability ≥ 0.99. IBD segments were inferred between the Kitka individual and the modern individuals in the THL_HCE dataset (Supplementary Notes and Data S4). The THL_HCE panel had 136,852 SNPs overlapping the non-missing, high probability positions in the imputed genome of KUU001. IBD segments were then inferred using IBIS with parameters mt 300, er 0.004, maxDist 0.1 and min_l 8.

To model the non-linear relationship between the spatial distributions of the Sámi-related and Kitka-related segments, we chose to use beta regression since it is a suitable method for modelling proportion data such as the IBD fractions, which take values between [0,1]. Beta regression does not allow for values exactly 0 or 1, which we accounted for by setting every 1 to 0.99999. Because we called only IBD segments longer than 8 cM, zero values are frequent in the data. To account for zero inflation, we modelled zero values explicitly with a hurdle model. The hurdle model was implemented in R with the package UHM using the function ZIHR [125]. We used longitude as a predictor in the model but omitted latitude due to the obvious correlation between these variables and Sámi ancestry, and since latitude was of little interest in the final analysis. We present key statistics and parameters of the model in Data S9.

### Stable isotope analysis

Each sampled skeletal element corresponds to a different time of formation (Table S2). Taking into account the exact sampling locations, the age representation of the premolar (FDI 35) samples spans ca. 3–7 years of age (enamel 3–4 y; dentine 3–7 y), while the samples from the third molar (FDI 48) represent material mineralised at ca. 9–18 years of age (enamel 9–13 y; root 13–18 y; [126–128]. Bone tissue remodels continuously through life. Thus, the rib specimen carries an average isotope signal recorded during the last few years prior to death. However, in the case of Sr isotope composition, unburnt bones are not considered reliable archives of in vivo signals, as they are known to be quickly overridden by Sr from the burial environment [129, 130]. The Sr isotope values of the rib are therefore representative of the characteristic ^87^Sr/^86^Sr values in the soil of the burial site.

The sampling and analysis of dental enamel, dentine and rib bone followed established standard procedures [131–133] with minor modifications. A more detailed description is provided in Supplementary Notes.

### Dietary modelling

Bayesian dietary modelling with FRUITS 3.0 [134] was performed on the measured stable carbon and nitrogen isotopic ratios to estimate relative contributions of terrestrial, freshwater and marine/brackish food groups within the diet of the individual. The diet was initially estimated to consist of three major food groups: terrestrial resources (TR; plants, animals, dairy products), freshwater fish (FF) and marine animals (MA). The dietary modelling procedure was based on the protocol by Oinonen et al. [135]. Particularly, the input data contained the measured stable carbon and nitrogen isotopic determinations, isotopic baseline data for TR, FF and MA food groups obtained mainly from the dedicated open-access δIANA database [136] – converted to represent isotopic ratios of food-group specific edible macronutrients – and food-group specific macronutrient fractions obtained from National Food Composition Database of Finland, FINELI (https://fineli.fi/fineli/en/index) and/or from National Nutrient Database, United States Department of Agriculture (https://fdc.nal.usda.gov/). Differing from the plain average of assumed food item values used by Oinonen et al. [135] for TR, here the nitrogen isotope values and macronutrient concentrations were weighted (80%) towards animal data, since a larger part of nitrogen comes from animal sources and due to enhanced animal food consumption at high latitudes [50]. Eventually, the modelling provided relative contributions of the food groups towards the consumer diet (αXX, where XX = TR, FF, MA or other selected food group).

To evaluate the sensitivity of the models towards carbon origin, subsets of marine (MA) dietary input data were assumed to correspond to potentially different geographical origins of the Barents Sea or White Sea. The isotopic baseline for the Barents Sea was obtained based on existing measurement data of marine animals from the region. However, as there was no isotopic data available of marine fauna from the White Sea, we relied on the measured difference of particulate organic carbon (POC) observed between the Barents Sea (ẟ^13^C_POC, Barents_ = -24.5 ± 0.9‰) and the White Sea (ẟ^13^C_POC, White_ = -29.0 ± 0.7‰). The lower ẟ^13^C_POC_ levels of the White Sea are due to terrestrial carbon inflow via rivers [68]. Subsequently, an offset of -4.5 ± 1.1‰ was adopted to convert the Barents Sea isotopic baseline for carbon to represent the White Sea. Further, we note that the isotopic signatures of Norwegian Sea POC resemble those of the Barents Sea [68] and the Bothnian Bay isotopic baseline [135] to those of the White Sea. Therefore, the model does not allow distinguishing between these areas, respectively. In addition, sensitivity analyses were also performed with dietary modellings using TR divided to terrestrial plants (TP) and terrestrial animal resources (TAR), and by assuming TAR containing mainly reindeer meat, as it has a distinctively high ẟ^13^C value due to lichen diet [137, 138]. The full food-group and macronutrient-specific isotopic baseline and macronutrient concentrations have been given in Tables S3 and S4.

## Supplementary Information

Supplementary Material 1.

Supplementary Material 2.

Supplementary Material 3.

## Data Availability

Raw sequencing data generated in this study are available in the European Nucleotide Archive under accession PRJEB88996.Genotype data used in the analyses are available in Poseidon Community Archive https://github.com/poseidon-framework/community-archive.The Finnish Biobank data analysed in this study is accessible via application procedure from the Biobank of Finnish Institute of Health and Welfare at http://www.thl.fi/biobank.
