# Supplementary material for: Bioarchaeological analysis illustrates the life of a 16th-century Sámi individual from Kitka, Kuusamo, northern Finland
Source: BMC Genomics. 2026 May 25;27:622. doi: 10.1186/s12864-026-12962-x (PMC13383393; doi:10.1186/s12864-026-12962-x)
Supplement: Supplementary file 1 — Supplementary Material 1. [file 12864_2026_12962_MOESM1_ESM.docx]

Stable isotope and dietary modelling data tables

Table S2. Modelled food group fractions (αTR, FF, MA) and protein and energy intakes (βProtein, Energy) for the Kitka individual samples. Uncertainties (1σ provided by the model) for the last digit of theintake value are given in parentheses. FDI35: premolar, FDI48: third molar. Age (years) corresponds to the age of formation for the analysed tooth/bone sample.

| **Sample** | **Age (years)** | **Source area** | **Terrestrial**  **αTR** | **Freshwater**  **αFF** | **Marine**  **αMA** | **Protein**  **βProtein** | **Energy**  **βEnergy** |
| --- | --- | --- | --- | --- | --- | --- | --- |
| FDI 35 | 3-7 | Barents Sea | 0.69(7) | 0.11(7) | 0.20(7) | 0.59(5) | 0.41(5) |
|  |  | White Sea | 0.67(8) | 0.07(6) | 0.26(9) | 0.58(5) | 0.42(5) |
| FDI 48 | 13-18 | Barents Sea | 0.80(7) | 0.04(4) | 0.17(7) | 0.48(7) | 0.52(7) |
|  |  | White Sea | 0.76(7) | 0.03(3) | 0.21(7) | 0.55(5) | 0.45(5) |
| rib | the last few years | Barents Sea | 0.77(6) | 0.04(4) | 0.19(6) | 0.55(5) | 0.45(5) |
|  |  | White Sea | 0.78(7) | 0.03(3) | 0.19(8) | 0.55(4) | 0.45(4) |

Table S3. Food-group and macro-nutrient specific isotopic baseline used in the dietary modellings. Due to weighting of terrestrial animal and plant values (see methods) uncertainties for terrestrial values have been estimated as standard errors of the weighted means, except for the Energy components, for which weighted standard deviation was used. The latter reflects better the large difference of carbon isotopic ratios of fats and carbohydrates. Standard errors of means have been used for FF and MA. TR = terrestrial resources, TP = terrestrial plants, TAR = terrestrial animal resources, FF = freshwater fish, MA = marine animals.

| **Food group** | **Food fraction** | **δ^13^C(‰)** | **±** | **δ^15^N(‰)** | **±** | **Notes** |
| --- | --- | --- | --- | --- | --- | --- |
| TR | Protein | -24.1 | 0.4 | 5.8 | 0.6 | animals 80% + plants 20% |
| TR | Energy | -29.6 | 1.8 |  |  | animals 80% + plants 20% |
| TR_reindeer_ | Protein | -22.4 | 0.6 | 6.0 | 0.6 | only reindeer 80% + plants 20% |
| TR_reindeer_ | Energy | -28.9 | 1.5 |  |  | only reindeer 80% + plants 20% |
| TP | Protein | -27.1 | 0.4 | 1.3 | 0.6 | plants 100% |
| TP | Energy | -28.3 | 1.7 |  |  | plants 100% |
| TAR | Protein | -23.4 | 0.4 | 6.9 | 0.5 | animals 100% |
| TAR | Energy | -30.2 | 1.9 |  |  | animals 100% |
| FF | Protein | -29.0 | 0.9 | 15.2 | 0.8 |  |
| FF | Energy | -36.0 | 1.1 |  |  |  |
| MA_Barents_ | Protein | -15.6 | 0.4 | 16.5 | 0.8 |  |
| MA_Barents_ | Energy | -22.6 | 0.4 |  |  |  |
| MA_White Sea_ | Protein | -20.1 | 0.4 | 16.5 | 0.8 |  |
| MA_White Sea_ | Energy | -27.1 | 0.4 |  |  |  |

Table S4. Macronutrient dry-mass and dry-mass carbon concentrations for the food groups. TR = Terrestrial resources (80% animal-derived and 20% plants), FF = Freshwater fish, MA = Marine animals, TR_reindeer_ = Terrestrial resources (80% reindeer-derived and 20% plants), TAR = Terrestrial animal resources, TP = Terrestrial plants. See Oinonen et al. (2020) for more details. The following carbon contents of the macronutrients were assumed to calculate carbon mass fractions: Proteins → 52.4 %, Fats → 76.9 %, Carbohydrates → 44.4 %. Energy fraction has been calculated by summing Fats and Carbohydrates fractions. For TR, based on estimates of Cordain et al. (2000) for high-latitude hunter-gatherer cultures, it has been assumed that terrestrial animal-derived food constitutes 80% of the diet and plant food 20% and, subsequently, the original values have been weighted accordingly. TR_reindeer_, the terrestrial animal-derived food of 80% constitutes solely reindeer. Standard Error of the Weighted Mean (SEWM) has been used as uncertainty estimate for the weighted results instead of Standard Error of the Mean (SEM) used for unweighted results.

| Food group →  ↓ Food fraction | TR | **SEWM_TR_** | FF | **SEM_FF_** | MA | **SEM_MA_** | **TR_reindeer_** | **SEWM_TR_** | TAR | **SEWM_TAR_** | TP | **SEWM_TP_** |
| --- | --- | --- | --- | --- | --- | --- | --- | --- | --- | --- | --- | --- |
| Proteins (wt%) | 55.6 | 4.9 | 95.2 | 1.8 | 75.4 | 11.5 | 71.2 | 6.5 | 63.5 | 5.9 | 21.9 | 4.2 |
| Fats (wt%) | 26.1 | 3.0 | 4.8 | 1.8 | 24.6 | 11.5 | 15.4 | 1.1 | 30.4 | 4.1 | 7.8 | 0.9 |
| Carbohydrates (wt%) | 19.5 | 5.6 | 0.0 | 0.0 | 0.0 | 0.0 | 14.6 | 7.4 | 6.0 | 3.9 | 77.0 | 4.4 |
| Energy(wt%) | 45.6 | 10.8 | 4.8 | 1.8 | 24.6 | 11.5 | 30.1 | 7.5 | 36.5 | 5.7 | 84.7 | 4.5 |
| Proteins (wtC%) | 29.2 | 2.6 | 49.9 | 0.9 | 39.5 | 6.0 | 37.3 | 3.4 | 33.3 | 3.1 | 11.5 | 2.2 |
| Fats (wtC%) | 20.1 | 2.3 | 3.7 | 1.4 | 18.9 | 8.8 | 11.9 | 0.8 | 23.4 | 3.2 | 6.0 | 0.7 |
| Carbohydrates (wtC%) | 8.6 | 2.5 | 0.0 | 0.0 | 0.0 | 0.0 | 6.5 | 3.3 | 2.7 | 1.7 | 34.2 | 2.0 |
| Energy (wtC%) | 28.8 | 4.0 | 3.7 | 1.4 | 18.9 | 8.8 | 18.4 | 4.6 | 26.1 | 4.1 | 40.1 | 2.1 |

Dietary modelling results


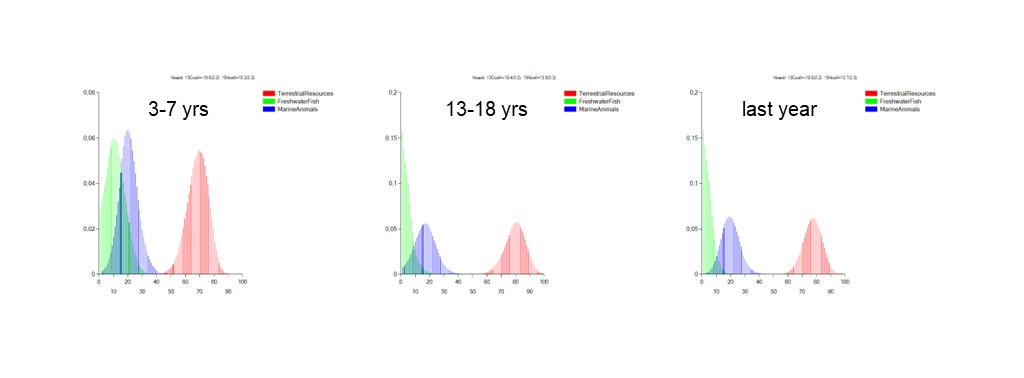
***Figure S7****. Dietary modelling results of noaidi individual of age 3-7 years (sample FDI35), 13-18 years (sample FDI48) and of the last year (sample rib) by assuming Barents Sea as the origin of marine dietary resources. Terrestrial animals (TR) = red, Freshwater fish (FF) = green, Marine animals (MA) = blue. Strong freshwater dietary component is observed corresponding to the age of 3-7 years.*


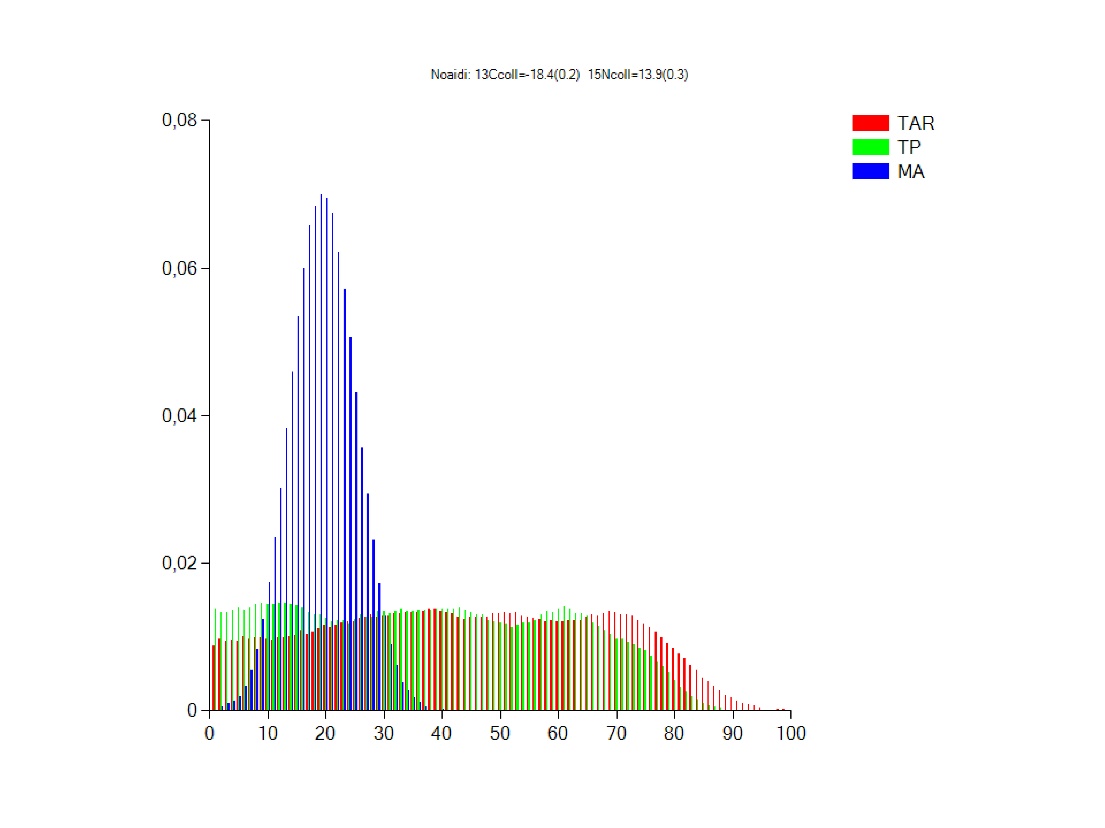


***Figure S8.*** *Dietary modelling results of noaidi individual of 13-18 years (sample FDI48) and of the last year (sample rib) by assuming Barents Sea as the origin of marine dietary resources and food groups of Terrestrial animal resources (TAR) = red, Terrestrial plants (TP) = green, Marine animals (MA) = blue. The distributions of TP and TAR are indistinguishable.*
