# Supplementary material for: Bioarchaeological analysis illustrates the life of a 16th-century Sámi individual from Kitka, Kuusamo, northern Finland
Source: BMC Genomics. 2026 May 25;27:622. doi: 10.1186/s12864-026-12962-x (PMC13383393; doi:10.1186/s12864-026-12962-x)
Supplement: Supplementary file 3 — Supplementary Material 3. [file 12864_2026_12962_MOESM3_ESM.docx]

Bioarchaeological analysis illustrates the life of a 16th-century Sámi individual from Kitka, Kuusamo, Northern Finland

Sanni Peltola^a,b,c,1^*, Ulla Nordfors^a,d,e,1^*, Laura Arppe^f^, Markku Oinonen^f^, Mika Sarkkinen^d,i^ , Miikka Voutilainen^c^, Kerttu Majander^g^, Thiseas C. Lamnidis^b^, Luca Traverso^b^, Johannes Krause^b^, Antti Sajantila^h,j^, Elina Salmela^a,b,d^, Päivi Onkamo^a^, Jussi-Pekka Taavitsainen^d^

1. University of Turku, Department of Biology, Turku, Finland
2. Department of Archaeogenetics, Max Planck Institute for Evolutionary Anthropology, Leipzig, Germany
3. Faculty of Biological and Environmental Sciences, University of Helsinki, Helsinki, Finland
4. University of Turku, Department of Archaeology, Turku, Finland
5. Museum Centre Vapriikki, Tampere, Finland
6. Finnish Museum of Natural History, University of Helsinki, Helsinki, Finland
7. Department of Environmental Sciences, University of Basel, Basel, Switzerland
8. Department of Forensic Medicine, University of Helsinki, Helsinki, Finland
9. Northern Ostrobothnia Museum, Oulu Museum and Science Centre, Oulu, Finland
10. Forensic Medicine Unit, Finnish Institute of Health and Welfare (THL)

^1^corresponding author

*equal contribution

#

[**Supplementary Notes 1**](#_u0jddn82ewl1)

[Burial context 1](#_61qsuop4mj7k)

[Pathogen screening and Finnish disease heritage genes 2](#_q1q9zvqya3hm)

[Reference data from Finnish Institute for Health and Welfare (THL) 3](#_dn9prlr2xve5)

[PCA populations 3](#_46v37db5zaqk)

[ADMIXTURE populations 4](#_d6tmtfs7c361)

[Stable isotope methods 4](#_c1g3zrj2des9)

[References 5](#_kww852x0u1u9)

[**Supplementary figures 7**](#_ooekuvilyrla)

# Supplementary Notes

## Burial context

The Kitka individual was found at Lehtoniemi in the modern village of Yli-Kitka, Kuusamo municipality, in 1970. The individual had been placed in a shallow grave near the ground surface in a north-south orientation and was accompanied by several artefacts: an axe, a knife, a silver belt buckle, a tin bird figurine with a cross motif, a silver coin carried as a pendant or amulet on the neck, and items interpreted as belonging to a Sámi noaidi’s drum kit: a drum hammer made of reindeer antler and copper rings [[1]](https://paperpile.com/c/J9WIhf/6WtO) (Fig. 1B). The coin is a Swedish 2 öre of John III, minted in 1573, and it dates the burial to the late 16th or early 17th century. The features and artefacts of the burial and its location on a former island suggest that it belonged to a Sámi noaidi [[1]](https://paperpile.com/c/J9WIhf/6WtO).

The noaidis played a significant role in the Sámi religion. They were ritual specialists responsible for maintaining balance in the world, and believed to be able to communicate with the spirit worlds. They used a drum in the rituals to move between worlds. In the post-medieval period, it was common for Sámi ritual specialists to incorporate Christian symbolism, such as crucifixes, into their religion and tools (e.g. [[2]](https://paperpile.com/c/J9WIhf/R72p). This can also be seen in the Kitka drum hammer that bears an orthodox cross [[1]](https://paperpile.com/c/J9WIhf/6WtO).

## Pathogen screening and Finnish disease heritage genes

We screened the double-stranded library of the Kitka individual (KUU001_ds) for potential pathogen sequences. Reads that did not map to the human genome were extracted for metagenomic analysis. Reads shorter than 30 bp were filtered out. The three algorithms from the HOPS package v0.35 [[3]](https://paperpile.com/c/J9WIhf/ccXo) were run manually. First, Malt was run with the following parameters against the full nucleotide database from NCBI: id=85, m=BlastN, at=SemiGlobal, top=1, supp=0.01, mq=100. The resulting rma6 files were sent to MaltExtract v1.7 with the following parameters: filter=def_anc, top=0.01, maxLength=0. Results were investigated with minPI values of 90.0 and 95.0. Finally, HOPS postprocessing was run to produce the plots.

We also screened the imputed genome of the Kitka individual for known disease-causing mutations for the following genetic conditions associated with the Finnish Disease Heritage, specifically ones that are more prevalent in the northeastern part of the country [[4]](https://paperpile.com/c/J9WIhf/j5dO): Salla disease [[5, 6]](https://paperpile.com/c/J9WIhf/17ct+oE3u), CNA2 (Pellegata et al. 2000), LCCS [[7]](https://paperpile.com/c/J9WIhf/BUjZ), CAAHD [[8]](https://paperpile.com/c/J9WIhf/XNvf), NPHS1 [[9]](https://paperpile.com/c/J9WIhf/faZv), LPI [[10]](https://paperpile.com/c/J9WIhf/TeaZ) and Nasu-Hakola disease [[11]](https://paperpile.com/c/J9WIhf/4RoY).

*Table S1. Genetic variants of the Kitka individual in positions associated with disease-causing genes in Finnish disease heritage.*

| **Disease name** | **Gene/Locus** | **dbSNP** | **Inheritance** | **Disease mutation allele** | **KUU001 alleles** |
| --- | --- | --- | --- | --- | --- |
| Congenital arthrogryposis with anterior horn cell disease (CAAHD) | *GLE1* | rs886063493 | Autosomal Recessive | G | AA |
| Cornea Plana 2 (CNA2) | *KERA* | rs121917858 | Autosomal Recessive | G | AA |
| Lethal congenital contracture syndrome (LCCS) | *GLE1* | rs386833693 | Autosomal Recessive | G | AA |
| Lysinuric protein intolerance (LPI) | *SLC7A7* | rs386833794 | Autosomal Recessive | T | CC |
| Nasu-Hakola disease | *TYROBP* | rs104894732 | Autosomal Recessive | C | TT |
| Finnish type of congenital nephrotic syndrome (NPHS1) | *NPHS1* | rs386833907 | Autosomal Recessive | T | CC |
| Salla disease | *SLC17A5* | rs80338794 | Autosomal Recessive | T | CC |

## Reference data from Finnish Institute for Health and Welfare (THL)

The genetic data from Finland used in this study originate from the National FINRISK Study (FINRISK), which is a population-based study conducted every five years since 1972 [[12]](https://paperpile.com/c/J9WIhf/lqfy). Genetic data from cohorts have been collected since 1992, along with information on the birthplace, parental birthplace, birth year and native language of the study participants, among other variables. In this study, we used genetic data from the 1992, 1997, 2002, 2007 and 2012 cohorts. We created two reference datasets: The first one, ‘THL_WGS’, contained the 1240k position pulldown from samples that had whole-genome sequencing data available. We performed a lift-over from hs38 to hs37 and merged the datasets with PLINK v1.90b3.29 [[13]](https://paperpile.com/c/J9WIhf/iURx). The final dataset included 281 individuals and 936,564 SNPs. These data were used in PCA, ADMIXTURE, and F statistics, but not in IBD analyses due to sparse geographic coverage, particularly in Lapland, our core area of interest. To obtain a better geographic coverage across Finland, we constructed a second dataset, ‘THL_HCE’, which was combined from genotyping array data from FINRISK cohorts. These included six genotyping batches, all genotyped with Illumina Human Core Exome genotyping arrays. These data were used only for the IBD analysis.

FINRISK genotyping batches were filtered as described in Nordfors et al. [[14]](https://paperpile.com/c/J9WIhf/Mjzg). The final THL_HCE dataset consisted of 3499 individuals and 230,708 SNPs.

Additionally, we included 19 Sámi-speaking individuals from the Health 2000 study (https://thl.fi/en/research-and-development/research-and-projects/health-2000-2011/health-2000-in-brief), genotyped in the Broad Institute with an unknown genotyping array. We included all these individuals irrespective of their parental birthplace. These individuals were only used in PCA projection and ADMIXTURE due to their low overlap with the 1240k SNP panel.

## PCA populations

Populations used for spanning the PCA were Abazin, Abkhasian, Adygei, Albanian, Altaian, Altaian_Chelkan, Ami, Armenian, Armenian_Hemsheni, Atayal, Avar, Azeri, Balkar, Balochi, Bashkir, Basque, BedouinA, BedouinB, Belarusian, Brahui, Besermyan, Bulgarian, Buryat, Cambodian, Canary_Islander, Chechen, China_Lahu, Chuvash, Circassian, Croatian, Cypriot, Czech, Dai, Darginian, Daur, Dolgan, Druze, Dungan, Enets, English, Estonian, Even, Evenk_FarEast, Evenk_Transbaikal, Ezid, Finnish, French, Gagauz, Georgian, Greek, Han, Hazara, Hezhen, Hungarian, Icelandic, Ingushian, Iranian, Italian_North, Italian_South, Itelmen, Japanese, Jew_Ashkenazi, Jew_Georgian, Jew_Iranian, Jew_Iraqi, Jew_Libyan, Jew_Moroccan, Jew_Tunisian, Jew_Turkish, Jew_Yemenite, Kabardinian, Kaitag, Kalash, Kalmyk, Karachai, Karakalpak, Karelian, Kazakh, Ket, Khakass, Khakass_Kachin, Khamnegan, Kinh, Korean, Koryak, Kumyk, Kurd, Kyrgyz_Kyrgyzstan, Kyrgyz_Tajikistan, Lak, Lebanese, Lezgin, Lithuanian, Makrani, Mala, Maltese, Mansi, Miao, Mongol, Mongola, Mordovian, Nanai, Naxi, Negidal, Nganasan, Nivh, Nogai_Astrakhan, Nogai_Karachay_Cherkessia, Nogai_Stavropol, Norwegian, Orcadian, Oroqen, Ossetian, Palestinian, Pathan, Polish, Russian_Archangelsk_Krasnoborsky, Russian_Archangelsk_Leshukonsky, Russian_Archangelsk_Pinezhsky, Russian, Saami.DG, Saami.WGA, Sardinian, Saudi, Scottish, Selkup, She, Shor_Khakassia, Shor_Mountain, Sicilian, Spanish, Spanish_North, Surui, Tabasaran, Tajik, Tatar_Kazan, Tatar_Mishar, Tatar_Siberian, Tatar_Siberian_Zabolotniye, Tatar_Tomsk.DG, Tatar_Volga.DG, Thai, Todzin, Tofalar, Tu, Tubalar, Tujia, Turkish, Turkish_Balikesir, Turkmen, Udmurt, Ukrainian, Ulchi, Uyghur, Uzbek, Veps, Xibo, Yakut, Yi, Yukagir, Kuusamo_Pohjois-Pohjanmaa, and KUU001_imputed.

## ADMIXTURE populations

Populations used in the ADMIXTURE analysis were Mbuti, Mbuti.DG, Yoruba, Hadza, Pima, Karitiana, Papuan, Onge, Mixe, Ami.DG, Atayal.DG, Han, Ulchi, Even, Mala, Brahmin_Tiwari, GujaratiB, Makrani, Brahui, Balochi, BedouinB, Kalash, Armenian, Cypriot, Greek, Croatian, Sardinian, Basque, Spanish, French, English, Scottish, Orcadian, Norwegian, Icelandic, Hungarian, Czech, Sorb, Polish, Ukrainian, Ukrainian_North, Belarusian, Lithuanian, Estonian, Finnish, Finnish.DG, Karelian, Veps, Mordovian, Russian, Russian_Archangelsk_Krasnoborsky, Russian_Archangelsk_Leshukonsky, Russian_Archangelsk_Pinezhsky, Chuvash, Komi_EBC, Udmurt, Mari.SG, Mari_EBC, Mansi, Mansi.DG, Selkup, Nganasan, Kuusamo_Pohjois-Pohjanmaa, Saami.DG, Saami.WGA, Saami_THLchip, Saami_Kola_EBC, Saami_Sweden_EBC, Finland_Levanluhta, Norway_Viking_o1.SG, Russia_Chalmny_Varre, KUU001_ds, KUU001_ss and KUU001_imputed.

## Stable isotope methods

Prior to sampling, the surface was removed with a dental burr fitted with a diamond-coated drill bit. Aliquots of homogenised sample powder were weighed for the extraction of bioapatite phosphate and collagen.

The ẟ^18^O value (VSMOW scale) of bioapatite phosphate was extracted as Ag_3_PO_4_. The pretreatment and precipitation of Ag_3_PO_4_ followed Wiedemann-Bidlack et al. [[15]](https://paperpile.com/c/J9WIhf/vsrY), but applying a longer precipitation time of 29 hours. Ag_3_PO_4_ crystals were pyrolysed at a temperature of 1400°C, and the ẟ^18^O value of the resulting CO gas was analysed on a Thermo Scientific Flash IRMS EA coupled to a Delta V Plus isotope ratio mass spectrometer. For normalisation, the known ẟ^18^O values of ANU sucrose (IAEA-CH6; 36.4‰), IAEA-601 (23.14‰) and two Ag_3_PO_4_ reference materials were used. The Ag_3_PO_4_ materials were AGPO-SCRI (14.58‰ [[16]](https://paperpile.com/c/J9WIhf/GZj4)) and an in-house material SJ-1 (5.56 ± 0.1‰; calibrated against USGS-80 and USGS-81 at the USGS Reston Stable Isotope Laboratory). As process quality controls over the analytical period, Nbs-120c phosphate rock material and an in-house enamel material ‘J-5’ were extracted, analysed and normalised alongside the unknowns. As a measure of process accuracy, Nbs-120c yielded a mean value of 21.9 ±0.10‰ (1σ). The external precision, as judged by repeats of Ag_3_PO_4_ reference materials and samples, was better than 0.3‰.

Collagen was extracted according to established methodologies based on Bocherens et al. [[17]](https://paperpile.com/c/J9WIhf/Tp99). The elemental content and isotopic composition of carbon and nitrogen were measured on an NC2500 elemental analyser coupled to a Thermo Scientific Delta V Plus isotope ratio mass spectrometer. The isotope data were normalised with a two-point calibration using international reference materials with known isotopic compositions (USGS-40, USGS-41). Extraction and IRMS measurement quality were monitored by analysing replicates of a caffeine powder and collagen extracted from an in-house bone reference material. The internal precision, evaluated from an in-house QC reference material, and duplicate sample measurements is ≤0.10 for both ẟ^13^C (VPDB) and ẟ^15^N (AIR).

For 87Sr/86Sr analysis, after acid digestion of the sample powder, Sr was separated by ion-exchange chromatography and its isotope ratio measured using multicollector inductively coupled plasma mass spectrometry (MC-ICP-MS; Thermo Scientific NeptunePlus) using internal standardisation and external calibration with bracketing isotope SRMs. The analysis was carried out by an ISO/IEC 17025 accredited laboratory, ALS Scandinavia AB, Luleå, Sweden. The results are mean values of two independent consecutive measurements, with a 2𝛔 standard deviation of 0.00004 or better.

## References

1. Kopisto A. Kuusamon lappalaishauta. Suomen Museo. 1971;78:64–72.
2. Svestad A. The impact of materiality on sámi burial customs and religious concepts. Fennoscandia archaeologica. 2011.
3. Hübler R, Key FM, Warinner C, Bos KI, Krause J, Herbig A. HOPS: automated detection and authentication of pathogen DNA in archaeological remains. Genome Biol. 2019;20:280.
4. Norio R. Finnish Disease Heritage II: population prehistory and genetic roots of Finns. Hum Genet. 2003;112:457–69.
5. Aula P, Autio S, Raivio KO, Rapola J, Thodén CJ, Koskela SL, et al. “Salla disease”: a new lysosomal storage disorder. Arch Neurol. 1979;36:88–94.
6. Harb JF, Christensen CL, Kan S-H, Rha AK, Andrade-Heckman P, Pollard L, et al. Base editing corrects the common Salla disease SLC17A5 c.115C>T variant. Mol Ther Nucleic Acids. 2023;34:102022.
7. Vuopala K, Herva R. Lethal congenital contracture syndrome: further delineation and genetic aspects. J Med Genet. 1994;31:521–7.
8. Vuopala K, Vuopala K, Ignatius J, Ignatius J, Herva R, Herva R. Lethal arthrogryposis with anterior horn cell disease. Hum Pathol. 1995;26:12–9.
9. Kestilä M, Lenkkeri U, Männikkö M, Lamerdin J, McCready P, Putaala H, et al. Positionally cloned gene for a novel glomerular protein--nephrin--is mutated in congenital nephrotic syndrome. Mol Cell. 1998;1:575–82.
10. Norio R, Perheentupa J, Kekomäki M, Visakorpi JK. Lysinuric protein intolerance, an autosomal recessive disease. A genetic study of 10 Finnish families. Clin Genet. 1971;2:214–22.
11. Hakola H. Neuropsychiatric and genetic aspects of a new hereditary disease characterized by progressive dementia and lipomembranous polycystic osteodysplasia. Acta Psychiatr Scand Suppl. 1972;232:1–173.
12. Borodulin K, Tolonen H, Jousilahti P, Jula A, Juolevi A, Koskinen S, et al. Cohort profile: The national FINRISK study. Int J Epidemiol. 2018;47:696–696i.
13. Purcell S, Neale B, Todd-Brown K, Thomas L, Ferreira MAR, Bender D, et al. PLINK: a tool set for whole-genome association and population-based linkage analyses. Am J Hum Genet. 2007;81:559–75.
14. Nordfors U, Peltola S, O’Sullivan RJ, Valtueña AA, Lamnidis TC, Majander K, et al. Archaeogenetics reveals fine-scale genetic continuity and patterns of kinship and health in medieval Finland. iScience. 2025;28:113086.
15. Wiedemann-Bidlack FB, Colman AS, Fogel ML. Phosphate oxygen isotope analysis on microsamples of bioapatite: removal of organic contamination and minimization of sample size. Rapid Commun Mass Spectrom. 2008;22:1807–16.
16. Halas S, Skrzypek G, Meier-Augenstein W, Pelc A, Kemp HF. Inter-laboratory calibration of new silver orthophosphate comparison materials for the stable oxygen isotope analysis of phosphates: New comparison materials for the stable O isotope analysis of phosphates. Rapid Commun Mass Spectrom. 2011;25:579–84.
17. Bocherens H, Billiou D, Patou-Mathis M, Bonjean D, Otte M, Mariotti A. Paleobiological implications of the isotopic signatures (13C,15N) of fossil mammal collagen in Scladina cave (Sclayn, Belgium). Quat Res. 1997;48:370–80.

# Supplementary figures


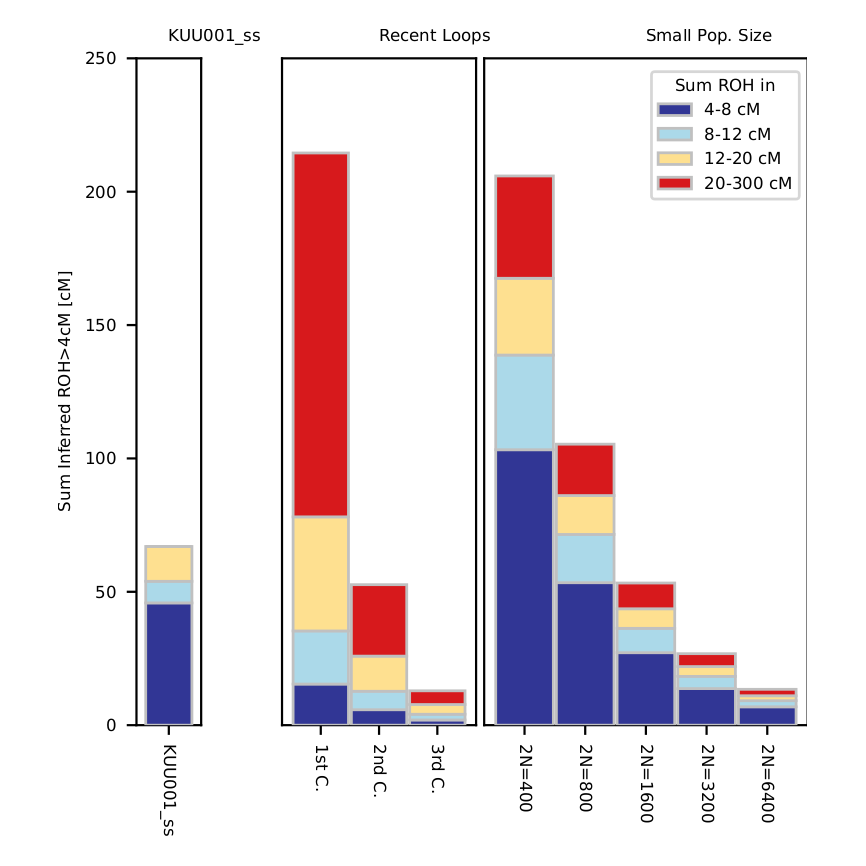


***Figure S1.*** *Runs of homozygosity (ROH) within the Kitka individual’s genome from hapROH analysis (left). Colours correspond to different ROH segment length bins. The two panels on the right show a simulated result for various scenarios of recent inbreeding (middle) and background relatedness (right).*


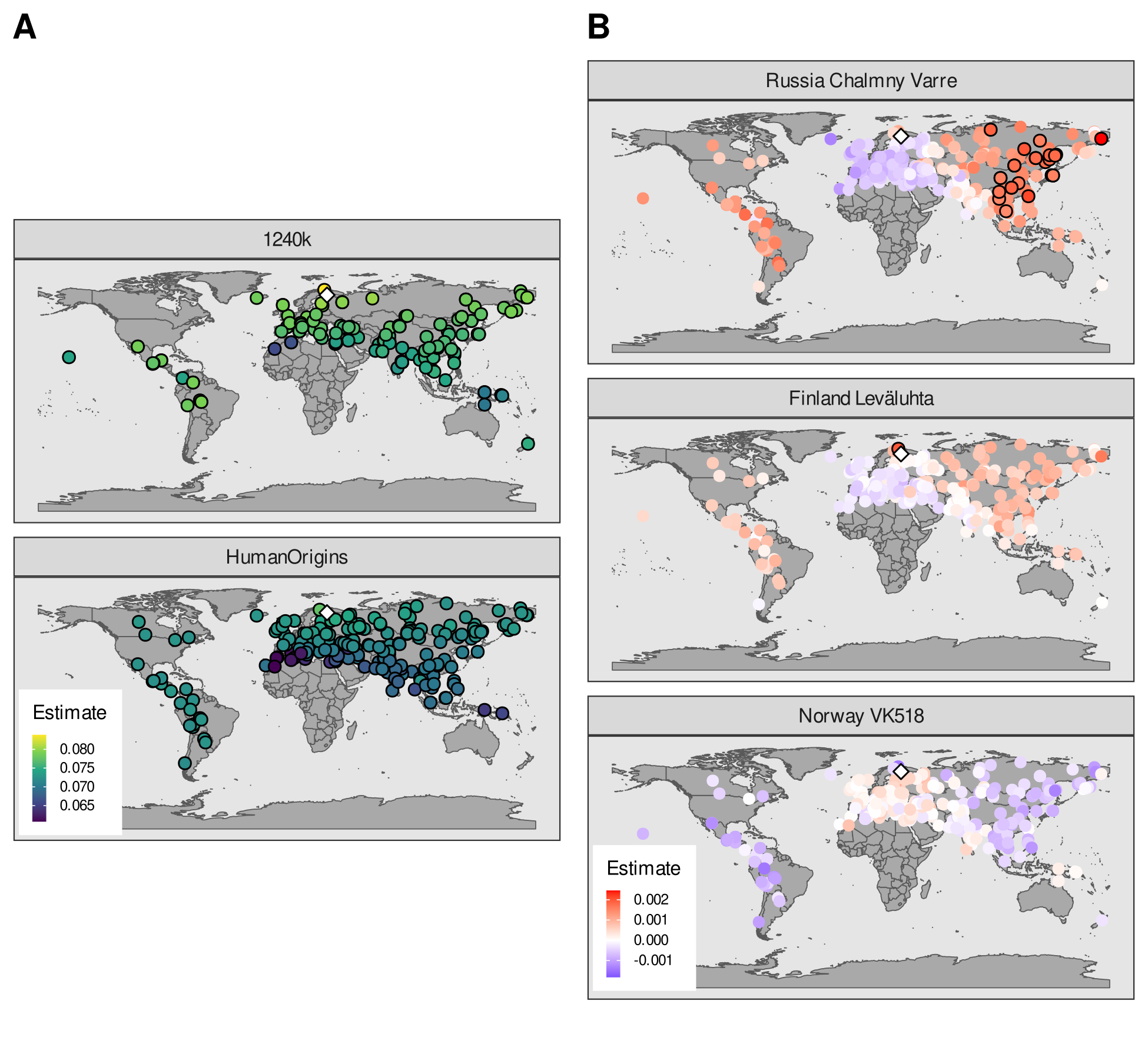
***Figure S2. A.*** *Outgroup F3 estimates between KUU001 and 399 present-day groups. Statistics are shown separately for groups genotyped only with the Human Origins panel. A white diamond marks the burial place of the Kitka individual.* ***B.*** *Test of symmetrical relatedness, f_4_(Mbuti, Test; X, KUU001_ss), where X is either Chalmny Varre, Leväluhta, or VK518, and Test is each 399 present-day groups as plotted on the maps. Outlined circles mark significantly non-zero estimates (|Z| ≥ 3)*


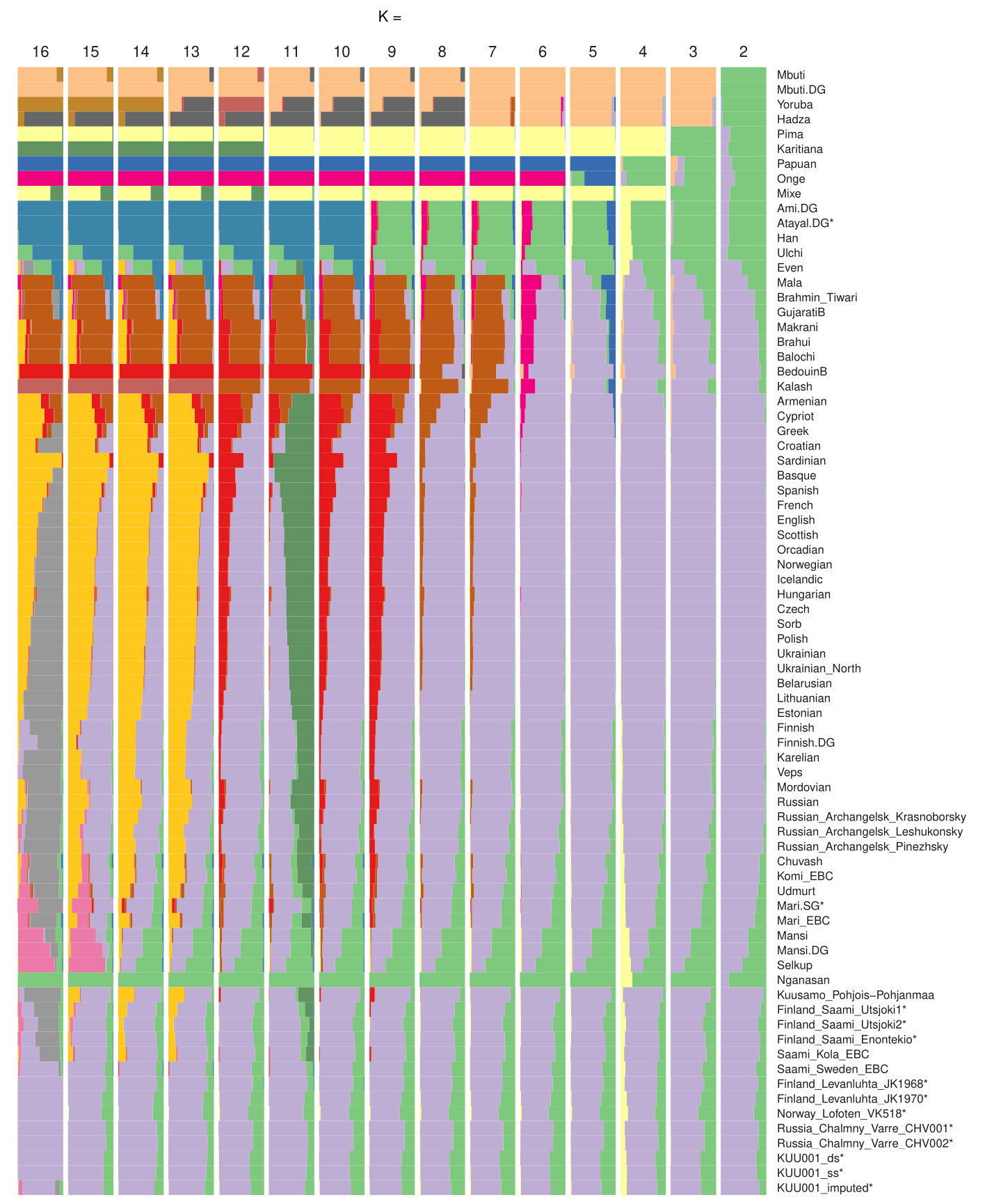


***Figure S3.*** *ADMIXTURE analysis. Bars without an asterisk show the average ancestry composition of the group.*


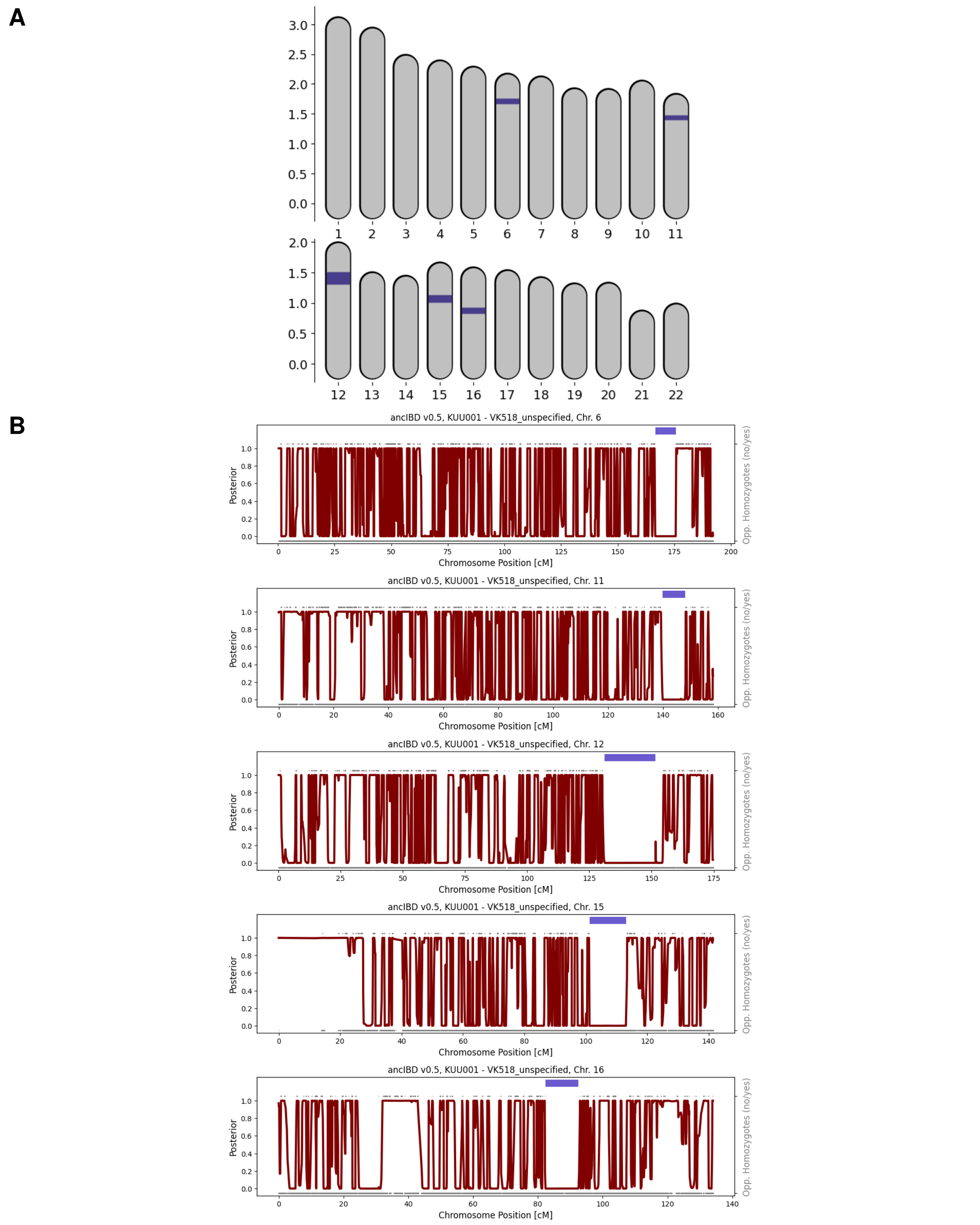
***Figure S4.*** *IBD sharing between KUU001 and VK518.* ***A.*** *IBD segments visualised in their chromosomal locations.* ***B.*** *Opposing homozygotes across the chromosomes where IBD segments were detected. Red line depicts the posterior probability from the ancIBD run, and purple lines highlight the discovered IBD segments.*


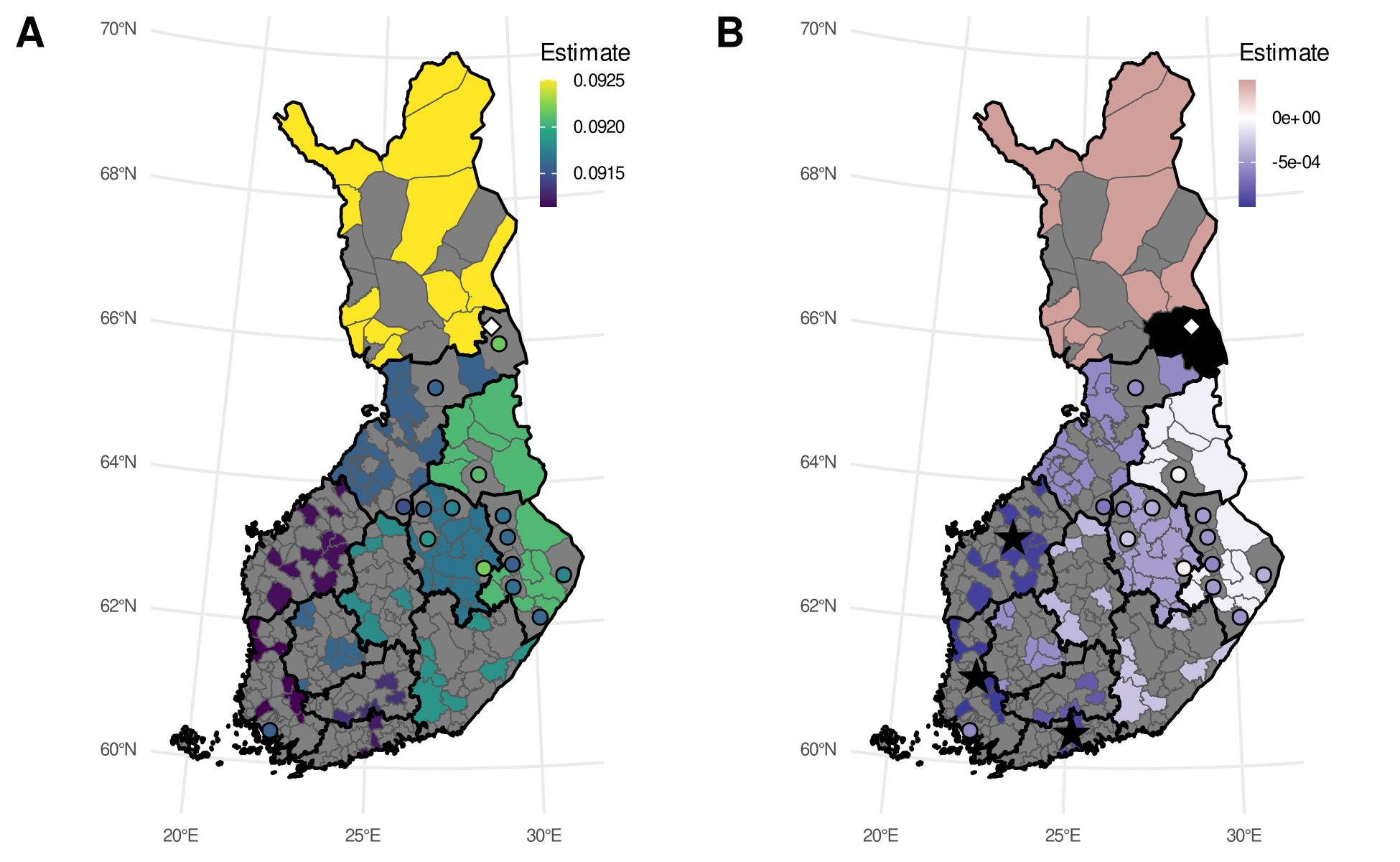
***Figure S5. A.*** *Outgroup F3 estimates between KUU001 and present-day Finns. Statistics are calculated per province, except for those municipalities that were represented by 5 or more individuals (plotted in circles). Municipalities with no data are shown in grey.* ***B.*** *F_4_ test for excess allele sharing between KUU001 and present-day Finns inhabiting the former Kitka region (black). Significant estimates of the test statistic f_4_(Mbuti, KUU001; Kitka, Test) are marked with asterisks.*


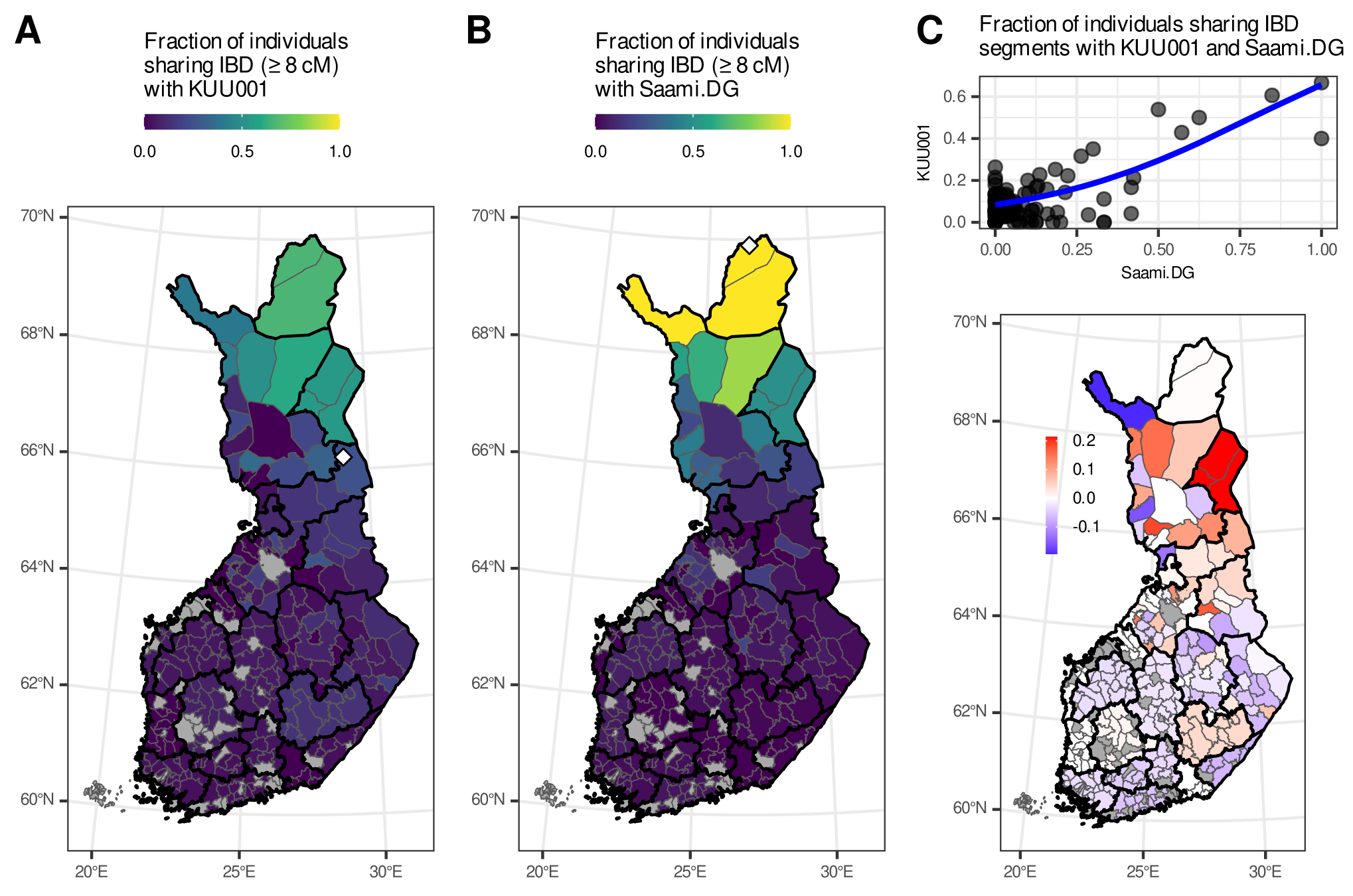
***Figure S6.*** *Fraction of individuals in each municipality sharing one or more IBD segments (≥8 cM) with* ***A.*** *KUU001 or* ***B.,*** *at least one of the two modern Sámi individuals from Utsjoki. Polygons with a black outline indicate areas of low data density, where the statistic has been averaged over a larger region than a single municipality. Municipalities with no data are shown in grey. White diamonds mark the location of the Kitka individual’s burial (A) and sampling locations of the two modern Sámi (B).* ***C.*** *A scatterplot of IBD sharing with KUU001 and modern Sámi in the analysis regions with predicted values of KUU001 IBD sharing from zero-inflated beta regression (blue line). Longitude is fixed to its mean for the purpose of plotting. The map shows residuals of the beta regression model.*
